# Host‐Driven Genetic Diversity of *Leptospira* in the Americas: A Continental Perspective

**DOI:** 10.1155/tbed/2456548

**Published:** 2026-01-16

**Authors:** Alejandro Suárez-Galaz, Sokani Sánchez-Montes, Marco Torres-Castro, Rodolfo Chan-Chan, Aarón Yeh-Gorocica, Wilson Moguel-Chin, Carlos I. Miranda-Caballero, Estefanía Grostieta, Alonso Panti-May, Hugo Ruiz-Piña, Roger Iván Rodríguez-Vivas, Anabel Cruz-Romero, Nadia F. Ojeda-Robertos, Enrique Reyes-Novelo

**Affiliations:** ^1^ Regional Research Center “Dr. Hideyo Noguchi”, Autonomous University of Yucatan, Merida, Mexico, uady.mx; ^2^ Faculty of Biological and Agricultural Sciences, University of Veracruz, Poza Rica–Tuxpan, Tuxpan de Rodriguez Cano, Mexico, uv.mx; ^3^ Faculty of Medicine, National Autonomous University of Mexico, Mexico City, Mexico, unam.mx; ^4^ Faculty of Veterinary Medicine and Zootecny, Autonomous University of Yucatan, Merida, Mexico, uady.mx; ^5^ Faculty of Veterinary Medicine and Zootecny, University of Veracruz, Veracruz, Mexico, uv.mx; ^6^ Academic Division of Agricultural Sciences, Autonomous University Juarez of Tabasco, Villahermosa, Mexico, ujat.mx

**Keywords:** America, genetic diversity, *Leptospira*, mammals, *SecY*

## Abstract

*Leptospira* is a genetically diverse genus of spirochetes comprising over 68 species, including several pathogenic taxa such as *L*. *interrogans*, *L*. *santarosai*, *L*. *noguchii*, and *L*. *weilii*. These bacteria infect a wide range of vertebrates, especially mammals, with infected animals serving as renal carriers that excrete the pathogen through urine. While rodents are the primary reservoirs for some species, multiple vertebrate orders participate in *Leptospira* transmission cycles in the Americas. This study aimed to assess and compare the genetic diversity of *Leptospira* populations across mammalian hosts throughout their distribution ranges in the Americas, exploring the influence of host interactions on bacterial diversity. Data for this study were obtained from two sources: (1) original screening of bats and rodents for pathogenic *Leptospira* and (2) partial gene sequences (*16S*, *LipL32*, and *SecY*) retrieved from GenBank, including sequences from human leptospirosis cases. A total of 321 animals were sampled (104 rodents and 217 bats), with an overall infection frequency of 12.1%. Positive samples were identified via BLAST as *L*. *interrogans*, *L*. *noguchii*, *L*. *santarosai*, *L*. *alexanderi*, and *L*. *weilii*. Genetic diversity metrics were calculated, and haplotype networks were constructed. Overall analyses revealed greater genetic diversity in bat *Leptospira* sequences, particularly in the *SecY* gene. In contrast, artiodactyls exhibited high intraspecific variation, suggesting a potential role in generating new *Leptospira* variants. Marsupials, rodents, and carnivores showed limited *Leptospira* diversity. These findings offer new insights into the evolutionary dynamics of *Leptospira* in the Americas and highlight the role of host ecology in shaping pathogen genetic diversity.

## 1. Introduction

The genus *Leptospira* comprises genetically diverse gram‐negative spirochetes [1, 2], encompassing more than 68 species grouped into two main clades: pathogenic (P) and saprophytic (S), each further divided into two subclades—P1 and P2 (pathogenic to humans and animals) and S1 and S2 (nonpathogenic species isolated from environmental sources) [3, 4]. The P1 subclade (P1+) includes eight highly virulent species—*L*. *interrogans*, *L*. *kirschneri*, *L*. *noguchii*, *L*. *santarosai*, *L*. *weilii*, *L*. *borgpetersenii*, *L*. *alexanderi*, and *L*. *mayottensis*—which are believed to have diverged from a common evolutionary node, with *L*. *interrogans* considered the most ancestral [3, 5].

In terms of geographic distribution, *L*. *interrogans*, *L*. *kirschneri*, and *L*. *borgpetersenii* are found worldwide. *Leptospira weilii* is commonly reported in Asia, Australia, and the Pacific region, while *L*. *noguchii* and *L*. *santarosai* are primarily documented in the Americas. *Leptospira mayottensis* is restricted to island nations in the Indian Ocean. Due to limited data, the distribution of *L*. *alexanderi* remains unknown [6].

Species within the P1+ subclade are responsible for significant health issues in humans and domestic animals. The most recent global estimates indicate that ~1.03 million people are infected with *Leptospira* annually. Of these cases, 873,000 progress to severe disease, and 59,000 are fatal [7, 8]. In the Americas, reported seroprevalence rates in 2017 reached ~28% [9].

Infections in companion animals, such as dogs and cats, pose a potential risk to public health due to the frequent close contact with humans. These animals may be exposed to environments contaminated with *Leptospira* where inadequate hygiene practices fail to prevent *Leptospira* transmission [10]. *Leptospira* infection in livestock, including cattle, pigs, and horses, poses a significant concern for the agricultural sector, as it can result in reproductive disorders in the animals and substantial economic losses [11, 12].

Pathogenic *Leptospira* species infect a wide range of vertebrates, primarily mammals, which serve as renal carriers and shed the bacteria through their urine [13]. Rodents are considered the primary natural reservoirs for *L*. *interrogans* and *L*. *borgpetersenii* [2]. However, several vertebrate orders—including Carnivora, Didelphimorphia, Chiroptera, Cingulata, and Afrosoricida—have been implicated in *Leptospira* transmission cycles worldwide, with the greatest concentration of studies having been conducted in the Americas [14].

In the Americas, Didelphimorphia (exclusive to this region), Carnivora, and Artiodactyla are among the most frequently studied mammalian orders. These are recognized as hosts for several pathogenic *Leptospira* species, including *L*. *interrogans*, *L*. *kirschneri*, *L*. *noguchii*, *L*. *santarosai*, *L*. *weilii*, *L*. *borgpetersenii*, and *L*. *alexanderi* [15–19]. Additionally, the Chiroptera order (bats) has growing attention due to the high diversity of pathogenic *Leptospira* species detected in these animals, both globally and, recently, in the Americas [20, 21].

In the Neotropical region of Mexico, rodents and bats are the most studied mammalian hosts of *Leptospira*. Wild and commensal rodents are hosts of *L*. *interrogans*, *L*. *kirschneri*, and *L*. *borgpetersenii* [22–26]. Bats from various trophic guilds host *L*. *interrogans*, *L*. *noguchii*, *L*. *santarosai*, and *L*. *weilii* [27–31], highlighting the high diversity of pathogenic *Leptospira* in these mammals in the region.

Given the broad host range of these bacteria, *Leptospira* has likely developed genetic adaptations that enable it to persist and colonize different host species avoiding their immune systems, promoting both interspecific and intraspecific genetic diversification [32, 33]. Evidence suggests that *Leptospira* undergoes genetic recombination [34] and exhibits haplotype‐level variation among species infecting different hosts [35–37]. However, it remains unclear whether the diversity of host species shows influence on the genetic diversity of pathogenic *Leptospira*.

Studying the genetic diversity of *Leptospira* provides insights into interspecific interactions with their hosts, population dynamics, colonization processes, migration, extinction, and the evolutionary mechanisms driving the emergence of genetic variants within this genus [38].

In *Leptospira*, several genes are used for accurate typing and taxonomic analysis [33]. In recent years, *LipL32* and *SecY* genes have been widely employed for population‐level studies [17, 39]. These markers, characterized by low mutation rates, high genetic variability, and neutral selection, are particularly valuable for genetic analyses, as they enhance inference and help describe traits specific to the populations under study [40, 41].

Given that pathogenic *Leptospira* species infect a broad range of mammalian hosts and that genetic traits are often shaped by environmental adaptation and inheritance, we would expect diversity in their population genetics across different host species in the Americas. The known genetic adaptations that enable *Leptospira* to infect different hosts and the interaction with their different immune systems, combined with the region’s rich diversity of pathogenic *Leptospira* across multiple mammalian orders [34], suggest that host‐specific interactions may drive genetic diversification.

In this context, the objective of this study was to assess the genetic diversity of *Leptospira* populations across the Americas and compare it throughout their distribution range, exploring the influence of host interactions on bacterial diversity.

## 2. Materials and Methods

### 2.1. Study Conceptualization and Design

To analyze the genetic diversity of *Leptospira* in several mammalian hosts across the Americas, two sources of information were used: (1) original data from partial gene sequences obtained from bats and rodents screened for pathogenic *Leptospira* species and (2) genetic data from partial gene sequences retrieved from public databases.

From July 2022 to June 2024, rodents and bats were captured at two sites in Yucatán, Mexico. Kidney tissue samples were collected for multilocus sequence typing (MLST) using the methodology described by Ahmed et al. [33], which enables the detection and characterization of pathogenic *Leptospira* species. In parallel, a targeted search was conducted in the NCBI GenBank database to retrieve sequences of pathogenic *Leptospira* species previously identified in bats and rodents from Yucatán, Mexico. This search was expanded to include the same *Leptospira* species identified in a broader range of mammalian hosts (Didelphimorphia, Rodentia, Chiroptera, Carnivora, Artiodactyla, and *Homo sapiens*) throughout the Americas.

### 2.2. Study Sites and Geographical Origin of *Leptospira* Sequences

**Figure 1:**
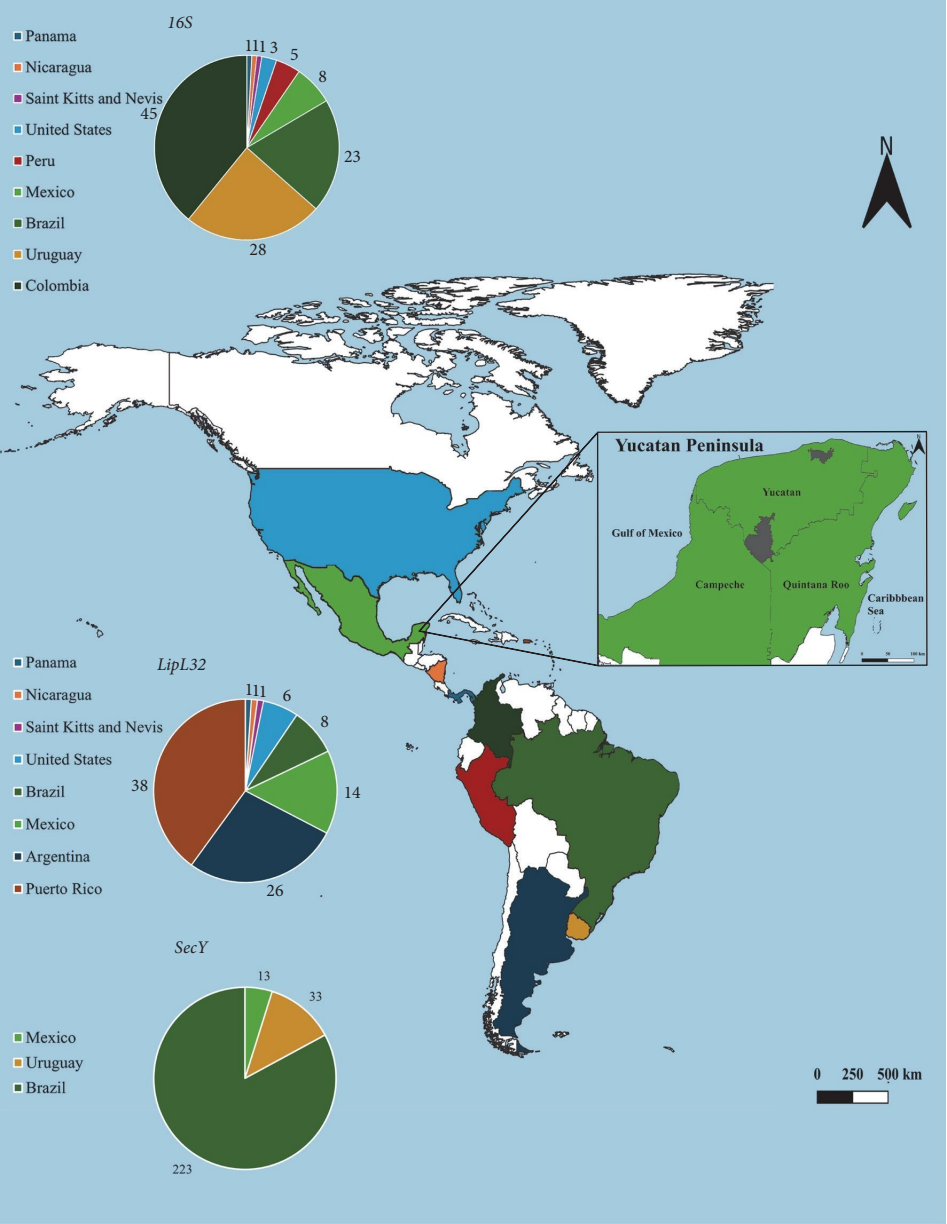
Geographic map of the Americas showing the territorial extent of countries from which sequences of pathogenic *Leptospira* species were obtained for genetic diversity analysis. The graphs represent the number of sequences per country. The inset highlights the Yucatán Peninsula, Mexico, and the specific municipalities (Panabá and Tekax) where field sampling was conducted.

Rodents and bats were captured in two municipalities in Yucatán: Panabá and Tekax (Figure 1). In Panabá, sampling was conducted at the “Santa María” ranch (21° 15’ 48.80” N, 88° 16’ 30.77” W), which has a warm subhumid climate with summer rains, an average annual temperature of 25.6 °C, and average annual precipitation of 975 mm. The site is located at an elevation of 8 m above sea level [42]. In Tekax, sampling was carried out at two locations: (1) “Grutas La Sartenejas” (20° 11’ 38.35” N, 89° 18’ 41.60” W) and (2) “Ecoparque Kaalmankal” (20° 12’ 21.92” N, 89° 18’ 18.01” W). Tekax also has a warm subhumid climate with summer rains, an average annual temperature of 25.7 °C, and an average annual precipitation of 950 mm. It is 37 m above sea level [43].

GenBank sequences of pathogenic *Leptospira* species used in this study were sourced from countries across the Americas and grouped into four regions: (1) North America (United States and Mexico), (2) Caribbean (Puerto Rico and Saint Kitts and Nevis), (3) Central America (Nicaragua and Panama), and (4) South America (Colombia, Peru, Brazil, Uruguay, and Argentina) (Figure 1).

### 2.3. Biological Material Collection

Rodents were captured using 120 Sherman traps (7.5 × 23 × 9 cm; HB Sherman Traps Inc., USA), placed linearly for two consecutive nights in two quadrants (60 traps per quadrant), with a spacing of 5 m between traps. The sampling effort totaled 2880 trap nights at site 1 and 1440 at site 2. The traps were baited with a mixture of oat flakes and artificial vanilla.

Bats were captured using three mist nets (6 × 2 m; Redes Ramirez, Mexico), placed parallel in the understory and operated for six consecutive hours (18:00–00:00). The sampling effort was 475.2 net hours at site 1 and 237.6 net hours at site 2. The captured bats were placed in drawstring cloth bags.

Rodents and bats were taken to the field stations for species identification and biological sampling. The rodents were processed as described by Reid [44], while the bats were processed as described by Medellín et al. [45]. The animals were anesthetized with isoflurane (Piramal Enterprises Limited, UK) and euthanized via intracardiac injection of sodium pentobarbital (90–210 mg/kg; Aranda, Mexico), according to Leary et al. [46]. A midline incision was made to collect the kidney fragments (cortex and medulla), which were preserved in 1.5 mL microcentrifuge tubes (Axygen, Mexico) filled with 96% ethanol and stored at −29 °C until processing.

### 2.4. Genomic DNA Extraction and Molecular Detection of *Leptospira*

Genomic DNA was extracted from the kidney tissue using Chelex‐100 (Bio‐Rad, USA) according to the methodology described by Tomazic et al. [47]. DNA concentration and purity were measured using a NanoDrop 2000 spectrophotometer (Thermo Scientific, USA).

Pathogenic *Leptospira* detection was performed via PCR targeting a 474‐bp fragment of the *LipL32* gene, using primers *LipL32F* (5′‐ATCTCCGTTGCACTCTTTGC‐3′) and *LipL32R* (5′‐ACCATCATCATCATCGTCCA‐3′) [33, 48]. Each 25 µL reaction contained 12.5 µL GoTaq Green Master Mix 2X (Promega, USA), 1 µL of each primer (2 µM), 8.5 µL nuclease‐free water, and 2 µL DNA template (100–250 ng). The thermal cycling protocol included 95 °C for 1 min followed by 35 cycles of 95 °C for 1 min, 55 °C for 30 s, and 72 °C for 1 min. The final 7‐min extension was at 72 °C [49].

Positive samples were then subjected to three more genes of the MLST scheme #3 [33], targeting *LipL41*, *rrs2*, and *SecY*. Gene‐specific amplification conditions and amplicon sizes are described by Ahmed et al. [33]. These housekeeping genes encode essential proteins and evolve slowly, making them suitable for phylogenetic and typing purposes [50].

PCR products were amplified using a Veriti 96‐well thermal cycler (Thermo Fisher Scientific, USA) and visualized via electrophoresis in 2% agarose gels stained with Midori Green Advance (Nippon Genetics Europe GmbH, Germany). Gels were documented using an OmniDOC system (Cleaver Scientific, UK; Supporting Information 1: Figure S1).

### 2.5. Infection Frequency and Confidence Interval Estimation

Infection frequency was estimated by taxonomic order and species of rodents and bats. Ninety‐five percent confidence intervals (CIs) were calculated using the Clopper–Pearson method via Quantitative Parasitology (QPWeb: http://www2.univet.hu/qpweb/qp10/) [51].

### 2.6. Sequencing and Species Identification

PCR–positive products (474–549 bp) were sent to Macrogen Inc. (Seoul, Korea) for Sanger sequencing. The resulting sequences were compared using BLAST‐N (Megablast algorithm) against validated *Leptospira* reference sequences in GenBank to determine homology and the infecting species in the rodents and bats from Yucatán, Mexico [52].

### 2.7. Retrieval of Pathogenic *Leptospira* Sequences From GenBank

For genetic diversity analysis, a curated search was conducted in GenBank for partial *16S*, *SecY*, and *LipL32* gene sequences of pathogenic *Leptospira* species (*L*. *interrogans*, *L*. *noguchii*, *L*. *santarosai*, *L*. *alexanderi*, and *L*. *weilii*) from mammalian hosts (Didelphimorphia, Rodentia, Chiroptera, Carnivora, and Artiodactyla) and human leptospirosis cases in the Americas. To ensure representation across the continent, sequences were categorized into four regions: North America, the Caribbean, Central America, and South America.

The search for gene fragment sequences was conducted using three different methods: (1) results of BLAST analysis of the sequences obtained in Mexico, (2) searching on the NCBI GenPop platform, and (3) searching on the GenBank platform for the identified species.

Keywords such as gene names (*16S*, *SecY*, and *LipL32*), pathogenic *Leptospira* species, hosts, and country were used in the search. Additionally, Boolean operators such as “AND,” “OR,” and “NOT” were implemented between each keyword. The origin time period for the sequences was from 1960 to 2024. Sequences without a collection date were also included if the data were complete.

A database was generated by downloading all sequences per gene. The sequence inclusion criteria were >90% coverage, >96% identity, and a length of >350 bp to ensure high‐confidence alignment and robust inference in diversity analyses. Sequences with duplicate accession numbers and extreme positions in the alignments were removed for the subsequent analysis.

The genes used for genetic analysis had different levels of resolution: *16S*, a highly conserved gene used for the identification of *Leptospira* species; *LipL32*, a highly conserved outer membrane gene among pathogenic *Leptospira* species; and *SecY*, which is highly polymorphic and capable of detecting intraspecific variation [33, 48, 49].

### 2.8. Global Alignment and Phylogenetic Tree Construction

Global alignments for the *16S*, *LipL32*, and *SecY* gene sequences were performed using the MUSCLE algorithm in MEGA X. The analysis included both the sequences obtained in this study (rodents and bats naturally infected) and the reference sequences retrieved from GenBank. Phylogenetic reconstruction was conducted using the maximum likelihood method in IQ‐TREE (http://www.iqtree.org/) [53], with 1000 bootstrap replicates to assess node support [54]. This approach allowed verification that the downloaded sequences corresponded to the target *Leptospira* species.

### 2.9. Genetic Diversity Analysis

Genetic diversity metrics were calculated for each gene using DnaSP v6.12.03 [55]. The following parameters were estimated: total number of sites, number of polymorphic sites (S), total number of mutations (η), number of haplotypes (*h*), haplotype diversity (Hd), nucleotide diversity (π). Neutrality tests, including Tajima’s *D* and Fu and Li’s *D* ^∗^ and *F* ^∗^, were also applied [56–59]. Diversity analyses were stratified by *Leptospira* species and mammalian host order.

### 2.10. Haplotype Network Construction

Haplotype networks were inferred using the Templeton, Crandall, and Sing (TCS) method [60], based on estimates of mutational steps per gene. Two networks were constructed: (1) haplotypes grouped by mammalian host order, and (2) haplotypes grouped by biogeographic region (North America, Caribbean Islands, Central America, and South America). All networks were generated and visualized using POPART v1.7 [61], incorporating previously described methods [60, 62, 63].

## 3. Results

### 3.1. Molecular Detection and Sequence Analysis of *Leptospira* From Animals Captured in Yucatán, Mexico

A total of 321 individual animals were captured, comprising 104 rodents (32.4%) and 217 bats (67.6%). The rodents were classified into three families, seven genera, and seven species, while the bats belonged to five families, 10 genera, and 10 species (Table 1).

**Table 1:** Frequencies of *Leptospira* infection by species of rodents and bats captured in Panabá and Tekax, Yucatán.

			Positive individuals to *Leptospira*/individuals analyzed (%)		Infection frequency by especies (IC 95%)
Order	Family	Species	Panabá	Tekax	
Rodentia	Heteromyidae	*Heteromys gaumeri*	2/22 (9.1)	1/3 (33.3)	12% (2.5–31.2)
	Cricetidae	*Ototylomys phyllotis*	3/42 (7.1)	0/6	6.3% (1.3–17.2)
		*Peromyscus yucatanicus*	0/16	0/1	—
		*Oryzomys couesi*	0/1	—	—
		*Reithrodontomys gracilis*	0/3	0/1	—
		*Sigmodon toltecus*	0/7	—	—
	Muridae	*Mus musculus*	0/1	—	—
		*Rattus rattus*	0/1	—	—
	Total		5/93 (5.4)	1/11 (9.1)	5.8% (2.1–12.1)

Chiroptera	Emballonuridae	*Peropteryx macrotis*	—	1/13 (7.7)	7.7% (0. 2–36)
	Phyllostomidae	*Diphylla ecaudata*	—	0/1	—
		*Glossophaga mutica*	0/8	26/75 (34.6)	31.3% (0.2–36)
		*Artibeus jamaicensis*	1/61 (1.6)	3/31 (9.7)	4.1% (0.8–11.4)
		*Carollia sowelli*	—	1/4 (25)	25% (0.6–80.6)
		*Dermanura phaeotis*	0/4	—	—
		*Sturnira parvidens*	1/16 (6.3)	0/1	6.2% (0.2–30.2)
	Mormoopidae	*Mormoops megalophylla*	—	0/1	—
	Molossidae	*Molossus nigricans*	0/1	—	—
	Vespertilionidae	*Myotis keaysi*	0/1	—	—
	Total		2/91 (2.2)	31/126 (14.3)	15.2% (10.7–20.7)

The overall frequency of *Leptospira* infection was 12.1% (39/321; 95% CI: 8.8–16.2). The infection prevalence was 5.8% in the rodents (6/104) and 15.2% in the bats (33/217). The infected rodent species included *Heteromys gaumeri* and *Ototylomys phyllotis*, while the infected bat species were *Peropteryx macrotis*, *Glossophaga mutica*, *Artibeus jamaicensis*, *Carollia sowelli*, and *Sturnira parvidens* (Table 1).

Gene fragments of *LipL32*, *LipL41*, *16S*, and *SecY* were successfully amplified as follows: 39 samples were amplified for both the *LipL32* and *SecY*, but only 28 samples were amplified for *16S* and 17 for *LipL41* fragments. Sequences were obtained only from 13 individuals for *LipL32*, *16S*, and *SecY* fragments: two rodents (*H*. *gaumeri* and *O*. *phyllotis*) and 11 bats (all *G*. *mutica*). BLAST analysis in the NCBI database revealed >99.6% identity with sequences corresponding to *L*. *interrogans*, *L*. *noguchii*, *L*. *santarosai*, *L*. *alexanderi*, and *L*. *weilii*. Among the bat sequences, four were identified as *L*. *noguchii*, four as *L*. *santarosai*, two as *L*. *alexanderi*, and one as *L*. *weilii*. The sequences obtained from the rodents were identified as *L*. *interrogans*.

### 3.2. Retrieved Sequences and Species Confirmation

A total of 95 *LipL32* sequences from 17 mammal species across eight countries were retrieved from GenBank. For the *16S* gene, 115 sequences were retrieved from 20 species across nine countries. The *SecY* dataset comprised 269 sequences from 30 mammal species distributed in three countries. Phylogenetic analyses based on maximum likelihood confirmed species‐level identifications and showed strong branch support (≥70), validating the taxonomic assignment of the reference sequences (Supporting Information 2: Figures S2, Supporting Information 3: Figures S3, and Supporting Information 4: Figures S4).

### 3.3. Genetic Diversity

Table 2 summarizes the genetic diversity parameters obtained for each gene and *Leptospira* species analyzed. Among the three genes, *SecY* displayed the highest number of polymorphic sites (*S* = 127), haplotypes (*h* = 52), the greatest haplotype diversity (Hd = 0.873), and nucleotide diversity (π = 0.078) values.

**Table 2:** Genetic diversity parameters and neutrality tests for the *SecY*, *LipL32*, and *16S* genes of *Leptospira* species identified in American hosts.

*Leptospira* species (*SecY*)						
Genetic diversity	*L. interrogans*	*L*. *noguchii*	*L*. *santarosai*	*L*. *alexanderi*	*L*. *weilii*	All species
Number of sequences	184	40	42	2	1	269
Total number of sites	393	408	408	408	408	408
S	20	28	43	3	—	127
η	20	29	46	3	—	159
*h*	17	11	21	2	—	52
Hd	0.740	0.810	0.950	1	—	0.873
π	0.005	0.021	0.027	0.500	—	0.078
Tajima’s *D*	−0.965	1.095	0.378	—	—	0.624
Fu and Li’s *D* ^∗^	−0.768	1.506	−0.880	—	—	1.156
Fu and Li’s *F* ^∗^	−1.015	1.618	−0.521	—	—	1.063

***Leptospira* species (*LipL32*)**						
**Genetic diversity**	***L. interrogans***	***L*. *noguchii***	***L*. *santarosai***	***L*. *alexanderi***	***L*. *weilii***	**All species**

Number of sequences	74	6	14	0	1	95
Total number of sites	443	443	443	—	443	443
S	—	4	12	—	—	21
η	—	4	12	—	—	21
*h*	1	3	5	—	—	6
Hd	—	0.773	0.769	—	—	0.287
π	—	0.004	0.010	—	—	0.019
Tajima’s *D*	—	0.355	0.273	—	—	−0.181
Fu and Li’s *D* ^∗^	—	0.071	0.382	—	—	0.059
Fu and Li’s *F* ^∗^	—	0.139	0.404	—	—	−0.036

***Leptospira* species (*16S*)**						
**Genetic diversity**	***L. interrogans***	***L*. *noguchii***	***L*. *santarosai***	***L*. *alexanderi***	***L*. *weilii***	**All species**

Number of sequences	101	6	6	1	1	115
Total number of sites	311	311	295	312	—	312
S	9	3	—	—	—	20
η	9	3	—	—	—	22
*h*	5	2	1	—	—	10
Hd	0.490	0.533	—	—	—	0.604
π	0.00281	0.005	—	—	—	0.009
Tajima’s *D*	−1.543	1.124	—	—	—	−1.101
Fu and Li’s *D* ^∗^	−3.194	1.395	—	—	—	−0.094
Fu and Li’s *F* ^∗^	−3.111^a^	1.406	—	—	—	−0.570

*Note*: S, number of polymorphic sites; *h*, number of haplotypes; Hd, haplotype diversity; π, nucleotide diversity; η, total number of mutations.

^a^Statistical significance.

Tajima’s *D* test for *SecY* suggested positive selection (*D* = 0.624), indicating a tendency toward increased genetic variation, whereas *LipL32* (*D* = −0.181) and *16S* (*D* = −1.101) revealed signatures of negative selection.

Species‐specific analysis of the *SecY* gene revealed that *L*. *santarosai* exhibited the greatest genetic diversity, with 43 polymorphic sites, 21 haplotypes, and 46 mutations, resulting in high haplotype (Hd = 0.950) and nucleotide (π = 0.027) diversity values. Although only two sequences were available for *L*. *alexanderi*, they were distinct from each other.

For the *LipL32* gene, *L*. *santarosai* again showed the highest diversity with 12 polymorphic sites and five haplotypes. However, haplotype and nucleotide diversity values were similar between *L*. *santarosai* (Hd = 0.769; π = 0.010) and *L*. *noguchii* (Hd = 0.773; π = 0.004). No genetic variation was observed among the 74 sequences of *L*. *interrogans* analyzed.

In contrast, analysis of the *16S* gene revealed that *L*. *interrogans* had the highest number of polymorphic sites (*S* = 9) and haplotypes (*h* = 5), likely due to the number of sequences (*n* = 101). No genetic variation was detected for *L*. *santarosai* in the *16S* dataset.

### 3.4. Genetic Diversity of *Leptospira* by Host Order

The analysis of *Leptospira* species and their associated hosts (Table 3) revealed greater genetic diversity among sequences identified in bats (Chiroptera) across all three genes analyzed. High haplotype diversity was observed in both the number of *Leptospira* sequences and the diversity of bat species sampled, particularly for *L*. *interrogans* and *L*. *noguchii*.

**Table 3:** Genetic diversity parameters and neutrality tests divided by host (mammalian order) and by gene (*SecY*, *LipL32* and *16S*) of *Leptospira*.

**Didelphimorphia**															
**Genetic diversity**	***L*. *interrogans***			***L*. *noguchii***			***L*. *santarosai***			***L*. *alexanderi***			***L*. *weilii***		
	***SecY***	***LipL32***	***16S***	***SecY***	***LipL32***	***16S***	***SecY***	***LipL32***	***16S***	***SecY***	***LipL32***	***16S***	***SecY***	***LipL32***	***16 S***

Number of host species	4	1	—	2	—	1	4	—	1	—	—	—	—	—	—
Number of sequences	5	1	0	2	0	1	4	0	1	0	0	0	0	0	0
Total number of sites	408	443	—	408	—	311	408	—	311	—	—	—	—	—	—
S	4	—	—	—	—	—	15	—	—	—	—	—	—	—	—
η	4	—	—	—	—	—	15	—	—	—	—	—	—	—	—
*h*	3	1	—	1	—	1	3	—	1	—	—	—	—	—	—
Hd	0.800	—	—	—	—	—	0.833	—	—	—	—	—	—	—	—
π	0.005	—	—	—	—	—	0.018	—	—	—	—	—	—	—	—
Tajima’s *D*	0.957	—	—	—	—	—	−0.847	—	—	—	—	—	—	—	—
Fu and Li’s *D* ^∗^	0.957	—	—	—	—	—	−0.847	—	—	—	—	—	—	—	—
Fu and Li’s *F* ^∗^	0.974	—	—	—	—	—	−0.866	—	—	—	—	—	—	—	—

**Rodentia**															
**Genetic diversity**	***L*** **.** ***interrogans***			***L*** **.** ***noguchii***			***L*** **.** ***santarosai***			***L*** **.** ***alexanderi***			***L. weilii***		
	***SecY***	***LipL32***	***16S***	***SecY***	***LipL32***	***16S***	***SecY***	***LipL32***	***16S***	***SecY***	***LipL32***	***16S***	***SecY***	***LipL32***	***16** **S***

Number of host species	8	4	4	2	—	—	1	1	1	—	—	—	—	—	—
Number of sequences	11	36	14	2	0	0	2	1	1	0	0	0	0	0	0
Total number of sites	408	443	311	408	—	—	408	443	311	—	—	—	—	—	—
S	3	—	3	—	—	—	2	—	—	—	—	—	—	—	—
η	3	—	3	—	—	—	2	—	—	—	—	—	—	—	—
*h*	4	1	3	1	—	—	2	1	1	—	—	—	—	—	—
Hd	0.600	—	0.615	—	—	—	1	—	—	—	—	—	—	—	—
π	0.001	—	0.003	—	—	—	0.004	—	—	—	—	—	—	—	—
Tajima’s *D*	−1.113	—	0.076	—	—	—	—	—	—	—	—	—	—	—	—
Fu and Li’s *D* ^∗^	−0.873	—	1.070	—	—	—	—	—	—	—	—	—	—	—	—
Fu and Li’s *F* ^∗^	−1.049	—	0.925	—	—	—	—	—	—	—	—	—	—	—	—

**Chiroptera**															
**Genetic diversity**	***L*. *interrogans***			***L*. *noguchii***			***L*. *santarosai***			***L*. *alexanderi***			***L*. *weilii***		
	***SecY***	***LipL32***	***16S***	***SecY***	***LipL32***	***16S***	***SecY***	***LipL32***	***16S***	***SecY***	***LipL32***	***16S***	***SecY***	***LipL32***	***16 S***

Number of host species	7	—	5	1	1	5	1	1	2	1	—	—	1	1	1
Number of sequences	12	0	5	4	4	3	4	2	2	2	0	0	1	1	1
Total number of sites	408	—	257	408	400	267	408	400	267	408	—	—	408	400	267
S	5	—	6	0	4	3	0	0	0	3	—	—	—	—	—
η	5	—	6	0	4	3	0	0	0	3	—	—	—	—	—
*h*	5	—	3	1	3	2	1	1	1	2	—	—	1	1	1
Hd	0.667	—	0.700	0	0.833	0.667	0	0	0	1	—	—	—	—	—
π	0.004	—	0.009	0	0.005	0.007	0	0	0	0.007	—	—	—	—	—
Tajima’s *D*	0.496	—	−1.145	—	−0.780	—	—	—	—	—	—	—	—	—	—
Fu and Li’s *D* ^∗^	−0.135	—	−1.145	—	−0.780	—	—	—	—	—	—	—	—	—	—
Fu and Li’s *F* ^∗^	0.029	—	−1.187	—	−0.720	—	—	—	—	—	—	—	—	—	—

**Carnivora**															
**Genetic diversity**	***L. interrogans***			***L. noguchii***			***L. santarosai***			***L. alexanderi***			***L. weilii***		
	***SecY***	***LipL32***	***16S***	***SecY***	***LipL32***	***16S***	***SecY***	***LipL32***	***16S***	***SecY***	***LipL32***	***16S***	***SecY***	***LipL32***	***16 S***

Number of hosts species	1	5	1	—	2	2	1	2	2	—	—	—	—	—	—
Number of sequences	46	10	37	0	2	2	1	3	2	0	0	0	0	0	0
Total number of sites	393	442	311	—	443	295	393	443	295	—	—	—	—	—	—
S	2	0	1	—	0	0	—	3	0	—	—	—	—	—	—
η	2	0	1	—	0	0	—	3	0	—	—	—	—	—	—
*h*	3	1	2	—	1	1	1	2	1	—	—	—	—	—	—
Hd	0.202	0	0.511	—	0	0	—	0.667	0	—	—	—	—	—	—
π	0.0005	0	0.002	—	0	0	—	0.004	0	—	—	—	—	—	—
Tajima’s *D*	−0.997	—	—	—	—	—	—	—	—	—	—	—	—	—	—
Fu and Li’s *D* ^∗^	−0.869	—	—	—	—	—	—	—	—	—	—	—	—	—	—
Fu and Li’s *F* ^∗^	−1.051	—	—	—	—	—	—	—	—	—	—	—	—	—	—

**Artiodactyla**															
**Genetic diversity**	***L. interrogans***			***L. noguchii***			***L. santarosai***			***L. alexanderi***			***L. weilii***		
	***SecY***	***LipL32***	***16S***	***SecY***	***LipL32***	***16S***	***SecY***	***LipL32***	***16S***	***SecY***	***LipL32***	***16S***	***SecY***	***LipL32***	***16S***

Number of host species	6	3	3	2	—	—	2	2	—	—	—	—	—	—	—
Number of sequences	108	28	41	32	0	0	31	7	0	0	0	0	0	0	0
Total number of sites	408	443	311	408	—	—	408	443	—	—	—	—	—	—	—
S	9	3	5	9	—	—	39	1	—	—	—	—	—	—	—
η	9	3	5	9	—	—	42	1	—	—	—	—	—	—	—
*h*	8	4	5	10	—	—	15	2	—	—	—	—	—	—	—
Hd	0.787	0.675	0.311	0.758	—	—	0.927	0.286	—	—	—	—	—	—	—
π	0.006	0.002	0.002	0.004	—	—	0.027	0.0007	—	—	—	—	—	—	—
Tajima’s *D*	1.097	0.589	−1.215	−0.471	—	—	0.297	−1.006	—	—	—	—	—	—	—
Fu and Li’s *D* ^∗^	0.553	0.958	1.114	0.773	—	—	−0.188	−1.048	—	—	—	—	—	—	—
Fu and Li’s *F* ^∗^	0.881	0.987	0.469	0.456	—	—	−0.033	−1.101	—	—	—	—	—	—	—

**Homo sapiens**															
**Genetic diversity**	***L. interrogans***			***L. noguchii***			***L. santarosai***			***L. alexanderi***			***L. weilii***		
	***SecY***	***LipL32***	***16S***	***SecY***	***LipL32***	***16S***	***SecY***	***LipL32***	***16S***	***SecY***	***LipL32***	***16S***	***SecY***	***LipL32***	***16S***

Number of sequences	2	—	4	—	—	—	—	—	—	—	—	1	—	—	—
Total number of sites	408	—	296	—	—	—	—	—	—	—	—	312	—	—	—
S	0	—	1	—	—	—	—	—	—	—	—	—	—	—	—
η	0	—	1	—	—	—	—	—	—	—	—	—	—	—	—
*h*	1	—	2	—	—	—	—	—	—	—	—	1	—	—	—
Hd	0	—	0.500	—	—	—	—	—	—	—	—	—	—	—	—
π	0	—	0.001	—	—	—	—	—	—	—	—	—	—	—	—
Tajima’s *D*	—	—	−0.612	—	—	—	—	—	—	—	—	—	—	—	—
Fu and Li’s *D* ^∗^	—	—	−0.612	—	—	—	—	—	—	—	—	—	—	—	—
Fu and Li’s *F* ^∗^	—	—	−0.478	—	—	—	—	—	—	—	—	—	—	—	—

*Note:* S, number of polymorphic sites; *h*, number of haplotypes; Hd, haplotype diversity; π, nucleotide diversity; η, total number of mutations.

For sequences identified in artiodactyls, *L*. *santarosai* showed the highest genetic diversity values based on the *SecY* gene (Hd = 0.927; *h* = 15; *S* = 39), followed by *L*. *noguchii* and *L. interrogans*. These sequences were obtained from two host species: *Bos taurus* and *Capra hircus*. Compared to other host orders (didelphimorphs, rodents, and carnivores), artiodactyl‐associated *L*. *santarosai* showed notably higher genetic variability.

In the order Didelphimorphia, the analysis of the *SecY* gene also revealed substantial haplotype diversity for both *L*. *interrogans* (Hd = 0.800) and *L*. *santarosai* (Hd = 0.833). Notably, although only three haplotypes were observed for *L*. *santarosai*, the number of polymorphic sites was higher (*S* = 15) than in *L*. *interrogans*. Neutrality tests (Tajima’s *D*) revealed contrasting selection patterns: *L*. *interrogans* exhibited signs of positive selection (*D* = 0.957), while *L*. *santarosai* showed evidence of negative selection (*D* = −0.847).

In contrast, low genetic diversity was detected in sequences from rodents (Rodentia) across all three genes. For example, analysis of *L*. *interrogans* sequences using the *LipL32* gene (*n* = 36) revealed no variation—only a single haplotype was identified, with no polymorphic sites or diversity detected.

Similarly, low diversity was observed in carnivores (Carnivora). An analysis of *L*. *interrogans* with *LipL32* (*n* = 10), which included sequences from five carnivore species, revealed no genetic diversity.

**Figure 2:**
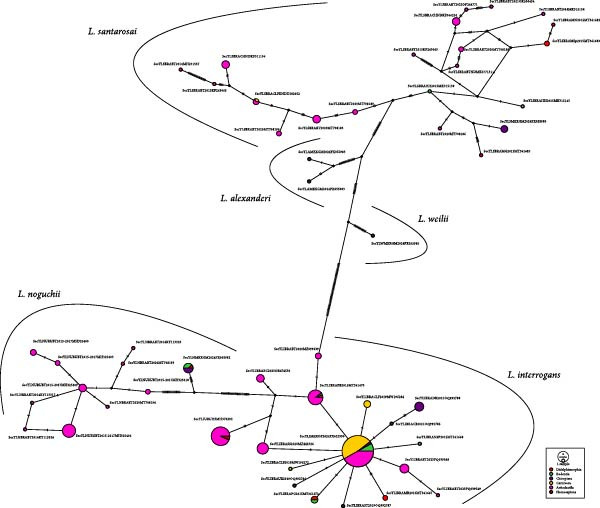
Haplotype network based on the *Leptospira SecY* gene fragment using sequences from American hosts (*n* = 269). The colors indicate the hosts (mammalian order) of *Leptospira*; the area of the circle is proportional to the number of sequences for each haplotype and its frequency in each host.

In addition to the mammal orders, sequences obtained from humans were also included. Although no significant genetic diversity was observed—likely due to the limited number of sequences per *Leptospira* species (Table 3)—haplotype network analysis revealed a shared *L*. *interrogans* haplotype between artiodactyls and humans(Figure 2 and Supporting Information 5: Figure S5), suggesting potential cross‐species transmission.

### 3.5. Haplotype Network

**Figure 3:**
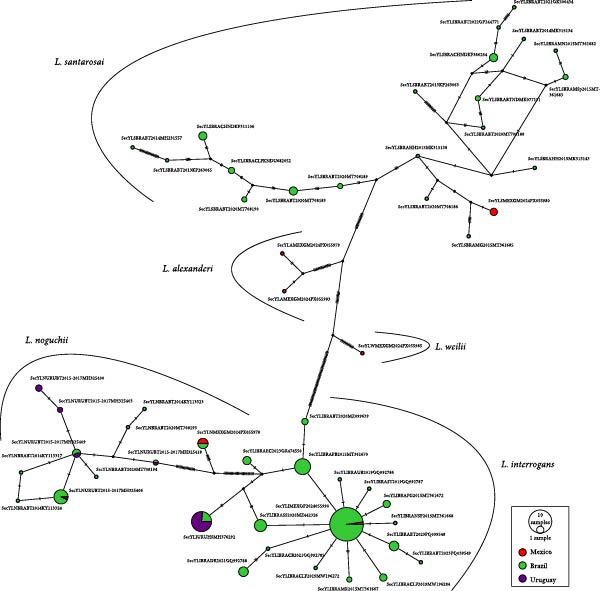
Haplotype network based on the *Leptospira SecY* gene fragment using sequences from American hosts (*n* = 269). The colors indicate the countries from which the haplotypes originate. The area of the circle is proportional to the number of sequences of each haplotype and its frequency in each country.

Haplotype network analysis of the *Leptospira SecY*, *16S*, and *LipL32* genes showed the genetic diversity and evolutionary relationships among the identified haplotypes (Figures 2 and 3, and complementary networks are in Supporting Information 4: Figure S4, Supporting Information 5: Figure S5, and Supporting Information 6: Figure S6). Specifically, the *Leptospira SecY* gene (Figure 2) revealed patterns of diversity, distribution, and evolutionary relationships among the identified haplotypes. The highest variability was observed in *L*. *santarosai* and *L*. *noguchii*, compared to *L*. *interrogans*. In particular, *L*. *santarosai* exhibited high haplotype diversity (*h* = 21; Hd = 0.950), with haplotypes distributed across all five mammalian orders. However, most haplotypes were order‐specific, except one shared between carnivores and artiodactyls.

For *L*. *noguchii*, haplotype diversity was concentrated primarily in artiodactyls, although one haplotype was shared among rodents, didelphimorphs, and bats. In contrast, *L*. *interrogans* showed lower overall haplotype diversity, with most haplotypes connected by one or two mutational steps. Notably, most *L*. *interrogans* sequences corresponded to a single dominant haplotype shared across carnivores, artiodactyls, rodents, and bats. Additional haplotypes were shared among two or more mammalian orders.

Hypothetical or unsampled ancestral haplotypes were observed across networks of all *Leptospira* species, including those with limited representation, such as *L*. *alexanderi* and *L*. *weilii*, which imply potentially extinct or under sampled populations in the network.

Finally, the geographic distribution of *SecY* haplotypes (Figure 3) showed a predominance of sequences originating from hosts in Brazil. For *L*. *interrogans* and *L*. *noguchii*, haplotypes were shared between Brazil and Uruguay, as well as between Brazil and Mexico. In contrast, the haplotypes of *L*. *santarosai*, *L*. *weilii*, and *L*. *alexanderi* identified in this study were found exclusively within single countries.

## 4. Discussion

Numerous genetic variants of *Leptospira* have been reported worldwide, associated with a wide range of mammalian hosts in the Americas [17, 36, 37], Europe [64–66], Africa [67, 68], and Asia [69]. These studies highlight the high interspecific and intraspecific genetic diversity of *Leptospira* [1, 70].

Most previous research has focused on a single taxonomic order of mammal hosts [37, 71]. Few studies have integrated sequences from public databases, such as GenBank, to analyze or contrast genetic diversity. In this study, we characterize the genetic diversity of *Leptospira* across multiple mammalian host orders using partial sequences from three genes.

Among the genes analyzed, *SecY* exhibited the highest polymorphism levels (Tables 2 and 3), consistent with its established use in taxonomic and genotyping studies due to its discriminatory power [72–74]. Intraspecific genetic diversity was highest in *L*. *santarosai*, followed by *L*. *noguchii* and *L*. *interrogans*, as supported by other studies using partial sequences obtained from humans, wild fauna, and domestic animals in the Americas [6, 36, 75–77].

In contrast, an analysis of the *LipL32* gene showed no variation in *L. interrogans* sequences. Although *L*. *interrogans* genetic diversity in the Americas is not well defined, some South American studies have reported low variability using partial sequences obtained from humans and *Rattus* spp. [36], while others have identified diverse genetic variants in rodents, marsupials, cattle, and pigs [37].

Our findings suggest that *L*. *santarosai* and *L*. *noguchii* may undergo recombination processes involving multiple host species. In *Leptospira*, recombination is frequently associated with genes regulating polysaccharide expression [34], which are critical components of the bacterial outer membrane and mediate interactions with host immune systems [78, 79]. Such processes may promote intraspecific recombination as part of *Leptospira* adaptation to different hosts or environmental conditions [34, 80].

In contrast, *L*. *interrogans* may exhibit a predominantly clonal structure in the Americas, consistent with its lower genetic diversity and reports of clonality in strains isolated in Brazil [81], the USA [82], and Thailand [83]. Nonetheless, further studies incorporating multiple genetic *Leptospira* markers in underrepresented regions such as Central and North America are necessary to clarify *Leptospira* diversity patterns continent‐wide.

Regarding host order‐specific patterns, high genetic variability was observed across all three *Leptospira* genes in sequences recovered from Chiroptera (Table 3). Bats are increasingly recognized as reservoirs for a wide range of infectious agents, including bacteria, viruses, and protozoa [84–86]. For *Leptospira*, several studies have demonstrated their role in hosting multiple pathogenic species [21, 87], including those identified in this study (*L*. *noguchii*, *L*. *santarosai*, *L*. *weilii*, and *L*. *alexanderi*).

Notably, *L*. *alexanderi* has been poorly documented in bats. Excepting a record from Colombia, our matching sequences for this species represent the second record for the Americas and the first time that a field study record sequences from this species in Mexico [20, 88]; however, this could be because *Leptospira* diversity studies in wildlife are scarce for Mexico [88].

The high local *Leptospira* species richness identified in the Yucatan studied animals is consistent with findings from nearby regions in Colombia [20, 89], which reinforces the role of bats in harboring several *Leptospira* species and maintaining multiple transmission cycles [90]. However, it remains unclear whether bats merely act as reservoirs or actively contribute to the generation of new genetic variants, as has been suggested for other pathogens such as *Trypanosoma cruzi* and *Bartonella* spp. [91, 92].

The highest levels of genetic diversity in this study were observed in *L*. *santarosai* partial sequences from artiodactyls, with haplotype network analysis further highlighting the intraspecific variability of both *L*. *santarosai* and *L*. *noguchii* within this host order (Figure 2). Compared to *L*. *interrogans* and *L*. *borgpetersenii*, available genetic information on *L*. *santarosai* and *L*. *noguchii* is more limited, primarily restricted to the Americas [6, 77]. Although these species are not strongly host‐specific, most of their known genetic variants have been described in South American cattle [71, 77–95].

Recent studies have reported *L*. *santarosai* genetic variants in urine and genital samples of cattle [96–98] and in the urine of equines from tropical Mexico [99]. *Leptospira noguchii*, while often considered incidental in livestock, may also adapt to the immune responses of bovine [11, 100]. The high intraspecific variation observed in both *L*. *santarosai* and *L*. *noguchii* may reflect these adaptive processes in artiodactyl hosts, including cattle, goats, pigs, and equines.

The interspecific and intraspecific diversity parameters identified here suggest that artiodactyls may have the potential to enhance the genetic diversity of *Leptospira*. These findings, consistent with earlier reports [95, 101], underscore the importance of cattle as key hosts capable of harboring diverse *Leptospira* genotypes with implications for both public and animal health [71]. Additional studies in Central and North America focusing on artiodactyl‐associated *Leptospira* isolates are needed to understand regional risks and genetic diversity.

High haplotype diversity was also observed in *L*. *interrogans* and *L*. *santarosai* sequences from didelphimorphs. Although the role of marsupials in *Leptospira* transmission cycles is not fully understood, studies in Brazil and Peru have documented the presence of *L*. *interrogans*, *L*. *noguchii*, *L*. *santarosai*, and *L*. *borgpetersenii* in kidney and urine samples [16, 102]. Other reports from the Americas have detected low‐level antibody responses (titers ≥ 800) in species such as *Didelphis virginiana*, *D*. *albiventris*, and *Marmosa micoureus* [102–105].

While *L*. *santarosai* has been identified in *D*. *albiventris*, phylogenetic analysis revealed no intraspecific variation [104]. More recently, *L*. *interrogans* sequences from marsupials have shown 100% identity with strains from humans, dogs, and synanthropic rodents in the same areas [99], suggesting possible interspecies transmission or shared environmental exposure.

Rodentia exhibited the lowest levels of *Leptospira* genetic diversity among the host orders analyzed, particularly for *L*. *interrogans*. Although this species is commonly associated with commensal rodents, such as those from the genus *Rattus* [106], it has also been documented in wild rodents, as in the present study, and in various regions across the Americas [16, 24, 98, 107].

As discussed, *L*. *interrogans* exhibits signs of clonal expansion, both at specific loci [81, 108] and at the whole‐genome level in urban rat populations (*Rattus* sp.) [82]. This limited genetic diversity may stem from a long evolutionary history of host‐pathogen coadaptation [109, 110]. It is possible that similar to *L*. *borgpetersenii*, *L*. *interrogans* exhibits minimal genetic diversity when colonizing rodent (reservoirs) kidneys, as has also been suggested for infections in artiodactyls [11, 100]. These genetic patterns may reflect the pathogen’s adaptation to specific host immune responses over time.

Despite this low diversity, further research into other pathogenic *Leptospira* species in rodents is warranted due to their epidemiological relevance as reservoirs in *Leptospira* transmission cycles.

A similar low genetic diversity was observed in all sequences analyzed, obtained from carnivores. Most sequences from this order were derived from domestic dogs (*Canis lupus familiaris*). Previous studies in the Americas have reported limited intraspecific genetic diversity in *L*. *interrogans*, *L*. *noguchii*, and *L*. *santarosai* in canine hosts [111], consistent with our findings. While dogs are known reservoirs of *L*. *interrogans* serovar Canicola [11], it remains unclear whether they contribute to the diversification of *Leptospira* species. However, current evidence suggests they do not, at least within the Americas.

Geographically, the majority of *Leptospira* sequences analyzed in this study originated from South America, followed by North and Central America.

Broadly, at a continental level, Brazil had the highest number of studies addressing on the molecular detection and sequencing of *Leptospira* [6, 14] mainly because an active epidemiological surveillance of Leptospirosis in livestock [96, 97, 112, 113] allowed the recording of a broader genetic diversity in isolates from production artiodactyls like cattle.

On the other side, the scarcity of molecular and serological studies in Central America and the Nearctic region of North America represents a significant knowledge gap for this analysis [9, 114]. To better understand *Leptospira* diversity at a continental scale, expanding surveillance and genetic analyses into these underrepresented areas is essential for future studies.

The observed genetic diversity in *Leptospira* species may be mediated by selective pressures resulting from adaptation to the host immune response [33, 115]. Likewise, adaptation to bacteria in animal hosts has been shown to occur through genetic mechanisms, including single‐nucleotide changes, genetic acquisitions or deletions, and genomic rearrangements [116, 117]. Even single‐nucleotide mutations can impact host tropism, as has been observed for other bacteria groups [118].

Likewise, in the present study, the collection date was not an inclusion criterion; analyzing the genetic diversity of sequences obtained from the 1990s decade to 2024, the diversity observed in some *Leptospira* species may be mediated by other factors such as the number of generations in time, allowing evolutionary processes over time due to the interaction with the immune system of different hosts [116, 117], as observed in epidemiological surveillance studies in other pathogens [119–124].

In this study, the amplification and/or sequencing of the *LipL41* gene was unsuccessful. Similar discrepancies have been reported in other studies using *Leptospira* MLST schemes [89, 125]. This problem can occur because MLST schemes are typically optimized for cultured isolates, where bacterial loads are higher and sequencing success improves [33, 70, 126]. Previous studies have compared MLST schemes between biological samples and bacterial cultures [125]; different biological samples such as serum, whole blood, and urine [127]; and even bacterial load between different cultures from clinical samples [125]. Results strongly suggest that the bacterial load of the samples determines the success of amplification or sequencing of all the genes in the MLST scheme.

Consequently, it is critical to develop and implement molecular protocols that enable the direct detection and typing of *Leptospira* from biological samples, such as tissues, blood, and urine, to gain a better understanding of their genetic structure, diversity, ecology, and evolutionary dynamics [126–130].

In recent decades, the presence of pathogenic *Leptospira* in different environments has been detected through culture and molecular methods [130]. Transmission occurs across a spectrum of ecological settings—from wild and rural areas to semirural and urban environments—and involves diverse host species [131, 132].

Although some studies have compared *Leptospira* prevalence in hosts across different ecological settings [16, 30, 90], it remains essential to approach *Leptospira* genetic diversity from an ecological perspective. Understanding host‐pathogen‐environment interactions at the local or regional levels is crucial for determining whether host diversity and ecological context influence the occurrence and variation of *Leptospira* genotypes.

From an epidemiologic perspective, the study of the genetic diversity of bacterial populations such as *Leptospira* implies important contributions to the study of zoonotic risks such as potential transmission routes between different hosts and human populations [50, 71] and risk factors related to the distribution of genetic variants [133] like the implementation of epidemiological surveillance strategies such as the detection and management of disease outbreaks [134, 135], the study of the evolution of highly virulent isolates [136, 137] veterinary health management, the design of new vaccines [138, 139], the improvement of vaccination strategies [140, 141], and field‐monitoring of the disease [142, 143].

From an ecological and evolutionary perspective, the results help to describe associations between hosts in their geographic distribution [144, 145] and to integrate phylogenetic evidence to understand the distribution and ancestry of bacterial populations with relevance for public and veterinary health [50].

This study contributes to such understanding by documenting part of the genetic diversity of *Leptospira* across multiple mammalian host orders in the Americas. Our results suggest that domesticated artiodactyls may be contributing to the increasing *Leptospira’*s genetic diversity, while bats appear acting as reservoirs of high haplotype richness. In contrast, marsupials, rodents, and carnivores suggest to limit intraspecific genetic diversity. These findings help to clarify essential factors that shape *Leptospira* species genetic diversity.

## Ethics Statement

This study was approved by the Bioethics Committee of the Faculty of Veterinary Medicine of the Autonomous University of Yucatán (document: CB‐CCBA‐D‐2022‐004). Animal capture and extraction were carried out with the scientific collection permit of the Mexican Ministry of Environment and Natural Resources (document: SPARN/DGVS/09663/23).

## Disclosure

All authors approved the final version of this article.

## Conflicts of Interest

The authors declare no conflicts of interest.

## Author Contributions

**Alejandro Suárez-Galaz**: conceptualization, data curation, formal analysis, investigation, methodology, software, writing – original draft preparation. **Sokani Sánchez-Montes**: conceptualization, funding acquisition, data curation, formal analysis, investigation, methodology, project administration, resources, software, validation writing – original draft preparation. **Marco Torres-Castro**: conceptualization, funding acquisition, project administration, resources, supervision, validation, visualization, writing – original draft preparation, writing – review and editing. **Rodolfo Chan-Chan**: investigation. **Aarón Yeh-Gorocica**: investigation. **Wilson Moguel-Chin**: investigation. **Carlos I. Miranda-Caballero**: investigation, methodology. **Estefanía Grostieta**: investigation, methodology, software. **Alonso Panti-May**: investigation, methodology, review and editing. **Hugo Ruiz-Piña**: review and editing. **Roger Iván Rodríguez-Vivas**: review and editing. **Anabel Cruz-Romero**: review and editing. **Nadia F. Ojeda-Robertos**: review and editing. **Enrique Reyes-Novelo**: conceptualization, funding acquisition, resources, supervision, validation, visualization, writing – original draft preparation, writing – review and editing.

## Funding

This research received no specific grant from any funding agency in the public, commercial, or not‐for‐profit sectors. Alejandro Suárez‐Galaz was supported by SECIHTI under scholarship number 001399.

## Supporting Information

Additional supporting information can be found online in the Supporting Information section.

## Supporting information


**Supporting Information 1** Figure S1: Agarose‐gel electrophoresis showing the amplified products of the *LipL32* (A) and *SecY* (B) gene fragments. (A) M: 100 bp DNA marker ladder; PCR controls; C+: positive control *L. interrogans* serovar Pomona DNA (474 bp); C−: negative control (without DNA); Lines 1–5, 7–8: negative samples; Lines 6, 9–24: positive samples. (B) M: 100 bp DNA marker ladder; PCR controls; C+: positive control *L. interrogans* serovar Pomona DNA (549 bp); C−: negative control (without DNA), Line 7: negative sample; Lines 1–6, 8–11: positive samples.

**Supporting Information 2** Figure S2: Phylogenetic tree generated using the maximum likelihood method with the sequences obtained from the nucleotide sequence database (GenBank) and the samples from the present study corresponding to the *Leptospira SecY* gene. The model for tree ensembly was the TN+F+G4. Bootstrap values (>0.5) are in the nodes. The scale bar indicates nucleotide substitutions per site. The diamonds highlight the *Leptospira* sequences identified in kidney tissue from the rodents and bats captured in the present study.

**Supporting Information 3** Figure S3: Phylogenetic tree generated using the maximum likelihood method with the sequences obtained from the nucleotide sequence database (GenBank) and the samples from the present study corresponding to the *Leptospira 16S* gene. The tree was generated using the K2P+I model. Bootstrap values (>0.5) are in the nodes. The scale bar indicates the nucleotide substitutions by site. The diamonds highlight the *Leptospira* sequences identified in kidney tissue from the rodents and bats captured in this study.

**Supporting Information 4** Figure S4: Phylogenetic tree generated using the maximum likelihood method with the sequences obtained from the nucleotide sequence database (GenBank) and the samples from this study corresponding to the *Leptospira LipL32* gene. The tree was generated using the K2P model. Bootstrap values (>0.5) are in the nodes. The scale bar indicates the nucleotide substitutions by site. The diamonds highlight the *Leptospira* sequences identified in kidney tissue from the rodents and bats captured in this study.

**Supporting Information 5** Figure S5: Haplotype network based on the *Leptospira 16S* gene fragment using sequences from American hosts (*n* = 115). The colors indicate (A) the hosts (mammalian order) and (B) the American region from which the haplotypes originate. The area of the circle is proportional to the number of sequences for each haplotype and its frequency, depending on the host or region.

**Supporting Information 6** Figure S6: Haplotype network based on the *Leptospira LipL32* gene fragment using sequences from American hosts (*n* = 95). The colors indicate (A) the hosts (mammalian order) and (B) the American region from which the haplotypes originate. The area of the circle is proportional to the number of sequences for each haplotype and its frequency, depending on the host or region. Colors indicate the *Leptospira* species, and the area of the circle is proportional to the number of sequences for each haplotype (host or region).

## Data Availability

The data that support the findings of this study are submitted to the National Center of Biotechnology Information (NCBI) GenBank database. The new sequences from Mexican mammals are under the following accession numbers: PV763579‐PV763589 for the *16S* gene, PX055969‐PX055977 for the *LipL32* gene, and PX055978‐PX055990 for the *SecY* gene.

## References

[1] Giraud-Gatineau A. , Nieves C. , and Harrison L. B. , et al.Evolutionary Insights Into the Emergence of Virulent *Leptospira* Spirochetes, PLOS Pathogens. (2024) 20, no. 7, 10.1371/journal.ppat.1012161, e1012161. 39018329 PMC11285912

[2] Levett P. N. , Leptospira, Manual of Clinical Microbiology, 2015, ASM Press, Washington DC, USA, 1028–1036.

[3] Vincent A. T. , Schiettekatte O. , and Goarant C. , et al.Revisiting the Taxonomy and Evolution of Pathogenicity of the Genus *Leptospira* Through the Prism of Genomics, PLOS Neglected Tropical Diseases. (2019) 13, no. 5, 10.1371/journal.pntd.0007270, 2-s2.0-85066853579, 5e0007270. PMC6532842 31120895

[4] Fernandes L. G. V. , Stone N. E. , and Roe C. C. , et al. *Leptospira sanjuanensis* sp. nov., a Pathogenic Species of the Genus Leptospira Isolated From Soil in Puerto Rico, International Journal of Systematic and Evolutionary Microbiology. (2022) 72, no. 10, 10.1099/ijsem.0.005560, 005560. 36260655

[5] Guglielmini J. , Bourhy P. , and Schiettekatte O. , et al.Genus-Wide *Leptospira* Core Genome Multilocus Sequence Typing for Strain Taxonomy and Global Surveillance, PLOS Neglected Tropical Diseases. (2019) 13, no. 4, 10.1371/journal.pntd.0007374, 2-s2.0-85066160202, e0007374. 31026256 PMC6513109

[6] Chinchilla D. , Nieves C. , and Gutiérrez R. , et al.Phylogenomics of *Leptospira santarosai*, a Prevalent Pathogenic Species in the Americas, PLOS Neglected Tropical Diseases. (2023) 17, no. 11, 10.1371/journal.pntd.0011733, e0011733. 37917733 PMC10645364

[7] Costa F. , Hagan J. E. , and Calcagno J. , et al.Global Morbidity and Mortality of Leptospirosis: A Systematic Review, PLOS Neglected Tropical Diseases. (2015) 9, no. 9, 10.1371/journal.pntd.0003898, 2-s2.0-84943161123, e0003898. 26379143 PMC4574773

[8] Picardeau M. , *Leptospira* and Leptospirosis, Leptospira Spp. Methods and Protocols, 2020, Springer US, New York, NY, 271–275. 10.1007/978-1-0716-0459-5_24 32632877

[9] Browne E. S. , Callefe J. L. R. , Jesus E. R. D. , Zeppelini C. G. , Cremonese C. , and Costa F. , A Systematic Review of the Geographic Distribution of Pathogenic *Leptospira* Serovars in the Americas, 1930–2017, Anais da Academia Brasileira de Ciências. (2022) 94, no. 3, 10.1590/0001-3765202220201026, e20201026. 36074401

[10] Ricardo T. , Azócar-Aedo L. I. , Previtali M. A. , and Monti G. , Seroprevalence of Pathogenic *Leptospira* Serogroups in Asymptomatic Domestic Dogs and Cats: Systematic Review and Meta-Analysis, Frontiers in Veterinary Science. (2024) 11, 10.3389/fvets.2024.1301959, 1301959. 38435371 PMC10904519

[11] Ellis W. A. , Animal Leptospirosis, Current Topics in Microbiology and Immunology. (2015) 387, 99–137, 10.1007/978-3-662-45059-8. 25388134

[12] Rocha B. R. , Martins G. , and Lilenbaum W. , An Historical View of the Experimental Leptospiral Infection in Ruminants, Comparative Immunology, Microbiology and Infectious Diseases. (2020) 73, 10.1016/j.cimid.2020.101532, 101532. 32980802

[13] Ko A. I. , Goarant C. , and Picardeau M. , *Leptospira*: The Dawn of the Molecular Genetics Era for an Emerging Zoonotic Pathogen, Nature Reviews Microbiology. (2009) 7, no. 10, 736–747, 10.1038/nrmicro2208, 2-s2.0-70349269503. 19756012 PMC3384523

[14] Cilia G. , Bertelloni F. , Albini S. , and Fratini F. , Insight Into the Epidemiology of Leptospirosis: A Review of *Leptospira* Isolations From “Unconventional” Hosts, Animals. (2021) 11, no. 1, 10.3390/ani11010191, 191. 33466962 PMC7830643

[15] Souza Rocha K. D. , Schupp De Sena Mesquita G. , and Silva Ferreira M. F. , et al.New Records of *Leptospira* spp. in Wild Marsupials and a Rodent in the Eastern Brazilian Amazon Through PCR Detection, Acta Amazonica. (2020) 50, no. 4, 305–308, 10.1590/1809-4392201903683.

[16] Medeiros L. D. S. , Braga Domingos S. C. , and Azevedo M. I. N. D. , et al.Small Mammals as Carriers/Hosts of *Leptospira* spp. in the Western Amazon Forest, Frontiers in Veterinary Science. (2020) 7, 10.3389/fvets.2020.569004, 569004. 33344523 PMC7738340

[17] Stone N. E. , Hall C. M. , and Ortiz M. , et al.Diverse Lineages of Pathogenic *Leptospira* Species are Widespread in the Environment in Puerto Rico, USA, PLOS Neglected Tropical Diseases. (2022) 16, no. 5, 10.1371/journal.pntd.0009959, e0009959. 35584143 PMC9154103

[18] Helman S. K. , Tokuyama A. F. , and Mummah R. O. , et al.Pathogenic *Leptospira* are Widespread in the Urban Wildlife of Southern California, Scientific Reports. (2023) 13, no. 1, 10.1038/s41598-023-40322-2, 14368. 37658075 PMC10474285

[19] Borges A. L. D. S. B. , Aymée L. , Carvalho-Costa F. A. , Lilenbaum W. , and Di Azevedo M. I. N. , Molecular Epidemiology of *Leptospira* spp. Serogroup Sejroe Associated With Chronic Bovine Leptospirosis, Veterinary Microbiology. (2024) 298, 10.1016/j.vetmic.2024.110238, 110238. 39216324

[20] Monroy F. P. , Solari S. , Lopez J. Á. , Agudelo-Florez P. , and Pelaez-Sanchez R. G. , High Diversity of *Leptospira* Species Infecting Bats Captured in the Uraba Region (Antioquia-Colombia), Microorganisms. (2021) 9, no. 9, 1897. 34576792 10.3390/microorganisms9091897 PMC8469583

[21] Matiz-González J. M. , Ballesteros-Ballesteros J. A. , and Hernández M. , et al.Genetic Diversity of P1/Pathogenic *Leptospira* Species Hosted by Bats Worldwide, Zoonoses and Public Health. (2024) 71, no. 5, 457–468, 10.1111/zph.13126. 38509439

[22] Torres-Castro M. A. , Gutiérrez-Ruiz E. , and Hernández-Betancourt S. , et al.First Molecular Evidence of *Leptospira* spp. in Synanthropic Rodents Captured in Yucatan, Mexico, Revue De Médecine Vétérinaire. (2014) 165, no. 7-8, 213–218.

[23] Torres-Castro M. , Cruz-Camargo B. , and Medina-Pinto R. , et al.Molecular Detection of Pathogenic *Leptospira* in Synanthropic and Wild Rodents Captured in Yucatán, México, Biomédica. (2018) 38, 51–58, 10.7705/biomedica.v38i3.3938, 2-s2.0-85055075025. 30184363

[24] Torres-Castro M. , Suárez-Galaz A. , and Yeh-Gorocica A. , et al.Identificación de *Leptospira interrogans* en *Ototylomys phyllotis* (Rodentia: Cricetidae) de Yucatán, México, Revista Cientifica de la Facultade de Veterinaria. (2024) 34, no. 2, e34383.

[25] Gutiérrez-Molina R. , Cruz-Romero A. , and Romero-Salas D. , et al.Molecular Evidence for the Presence of *Leptospira borgpetersenii* in Synanthropic Rodents in the Nautla Region, Veracruz, Mexico, Therya. (2019) 10, no. 2, 171–174, 10.12933/therya-19-723, 2-s2.0-85068770220.

[26] Rodríguez-Rojas J. J. , Rodríguez-Moreno Á. , Sánchez-Casas R. M. , and Hernández-Escareño J. J. , Molecular Detection of *Leptospira interrogans* and *Borrelia burgdorferi* in Wild Rodents From Mexico, Vector-Borne and Zoonotic Diseases. (2020) 20, no. 11, 860–863, 10.1089/vbz.2019.2600. 32639187

[27] Ballados-González G. , Sánchez-Montes S. , and Romero-Salas D. , et al.Detection of Pathogenic *Leptospira* Species Associated With Phyllostomid Bats (Mammalia: Chiroptera) From Veracruz, Mexico, Transboundary and Emerging Diseases. (2018) 65, no. 3, 773–781, 10.1111/tbed.12802, 2-s2.0-85046088273. 29318786

[28] Torres-Castro M. , Febles-Solís V. , and Hernández-Betancourt S. , et al. *Leptospira* Patógenas en Murciélagos de Campeche y Yucatán, México, Revista MVZ Córdoba. (2020) 25, no. 2, 10.21897/rmvz.1815, e1815.

[29] Torres–Castro M. , Alonso Panti–May J. , and MacSwiney González M. C. , et al.Detección de *Leptospira* spp. en Murciélagos de la Península de Yucatán, México, Revista Científica de la Facultade de Veterinaria. (2023) 33, no. 2.

[30] Suárez-Galaz A. , Reyes-Novelo E. , and Hernández-Betancourt S. , et al.Study on the Relation of the Characteristics of the Capture Sites With the *Leptospira* spp. Occurrence in Bats and Rodents From Yucatan, Mexico, Acta Tropica. (2024) 249, 10.1016/j.actatropica.2023.107072, 107072. 38008370

[31] Chong-Guzmán L. A. , Aréchiga-Ceballos N. , and Ballados-Gonzáles G. G. , et al. *Leptospira interrogans* Associated With the Common Vampire Bat (*Desmodus rotundus*) From the Neotropical Region of Mexico, Microbiology Research. (2025) 16, no. 2, 10.3390/microbiolres16020043, 43.

[32] Bulach D. M. , Zuerner R. L. , and Wilson P. , et al.Genome Reduction in *Leptospira borgpetersenii* Reflects Limited Transmission Potential, Proceedings of the National Academy of Sciences. (2006) 103, no. 39, 14560–14565, 10.1073/pnas.0603979103, 2-s2.0-33749249203. PMC1599999 16973745

[33] Ahmed N. , Devi S. M. , and De los Á Valverde M. , et al.Multilocus Sequence Typing Method for Identification and Genotypic Classification of Pathogenic *Leptospira* Species, Annals of Clinical Microbiology and Antimicrobials. (2006) 5, 1–10, 10.1186/1476-0711-5-28, 2-s2.0-34547645281. 17121682 PMC1664579

[34] Mejía L. , Prado B. , Cárdenas P. , Trueba P. , and González-Candelas F. , The Impact of Genetic Recombination on Pathogenic *Leptospira* , Infection, Genetics and Evolution. (2022) 102, 10.1016/j.meegid.2022.105313, 105313. 35688386

[35] Piredda I. , Ponti M. N. , and Palmas B. , et al.Molecular Typing of Pathogenic *Leptospira* Species Isolated From Wild Mammal Reservoirs in Sardinia, Animals. (2021) 11, no. 4, 10.3390/ani11041109, 1109. 33924303 PMC8069414

[36] Delgado M. A. , Cáceres O. A. , Calderón J. E. , Balda L. , Sotil G. , and Céspedes M. J. , New Genetic Variants of *Leptospira* spp. Characterized by MLST From peruvian Isolates, Journal of Tropical Medicine. (2022) 1, 1–13, 10.1155/2022/4184326, 4184326. PMC9553527 36249734

[37] Di Azevedo M. I. N. , Verde R. D. S. , and Ezepha C. , et al.Genetic Evidence for a Potentially New Pathogenic *Leptospira* sp. Circulating in Bats From Brazilian Amazon, Transboundary and Emerging Diseases. (2023) 1, 1–11, 10.1155/2023/9677047, 9677047. PMC12016835 40303746

[38] Robinson D. A. , Feil E. J. , and Falush D. , Bacterial Population Genetics in Infectious Disease, 2010, John Wiley & Sons.

[39] Sykes J. E. , Gamage C. D. , Haake D. A. , and Nally J. E. , Understanding Leptospirosis: Application of State-of-the-Art Molecular Typing Tools With a One Health Lens, American Journal of Veterinary Research. (2022) 83, no. 10, 10.2460/ajvr.22.06.0104. 35986911

[40] Porras-Hurtado L. , Ruiz Y. , Santos C. , Phillips C. , Carracedo A. , and Lareu M. V. , An Overview of STRUCTURE: Applications, Parameter Settings, and Supporting Software, Frontiers in Genetics. (2013) 4, 10.3389/fgene.2013.00098, 2-s2.0-84883521771, 98. 23755071 PMC3665925

[41] Hamilton M. B. , Population Genetics. Genotype Frequencies, 2021, 2nd edition, John Wiley & Sons, 9–51.

[42] Secretaria de Fomento Económico y Trabajo (SEFOET) , Panabá, Yucatán, 2020, Accessed April 14, 2023 http://www.sefoet.yucatan.gob.mx/secciones/ver/panaba.

[43] Secretaria de Fomento Económico y Trabajo (SEFOET). , Tekax, Yucatán, 2020, Accessed April 14, 2023 http://www.sefoet.yucatan.gob.mx/secciones/ver/tekax.

[44] Reid F. , A Field Guide to the Mammals of Central America and Southeast Mexico, 1997, Oxford University Press.

[45] Medellín R. A. , Arita H. T. , and Sánchez O. , Identificación de Los Murciélagos de México: Clave de Campo, 2008, Asociación Mexicana de Mastozoología, 26–63.

[46] Leary S. , Underwood W. , and Anthony R. , et al.AVMA Guidelines for the Euthanasia of Animals: Edition, 2020, American Veterinary Medical Association.

[47] Tomazic M. L. , Hamer M. , and Bustos C. P. , et al.Use of Chelex-100 for the Molecular Diagnosis of Five Animal Pathogens, Fave. Sección Ciencias Veterinarias. (2021) 20, no. 1, 26–33, 10.14409/favecv.v20i1.9724.

[48] Tansuphasiri U. , Chanthadee R. , Phulsuksombati D. , and Sangjun N. , Development of a Duplex-Polymerase Chain Reaction for Rapid Detection of Pathogenic *Leptospira* , The Southeast Asian Journal of Tropical Medicine and Public Health. (2006) 37, no. 2, 297–308. 17124990

[49] Vital-Brazil J. M. , Balassiano I. T. , Oliveira F. S. de , Costa A. D. de S. , Hillen L. , and Pereira M. M. , Multiplex PCR-Based Detection of *Leptospira* in Environmental Water Simples Obtained From a Slum Settlement, Memórias do Instituto Oswaldo Cruz. (2010) 105, no. 3, 353–355, 10.1590/S0074-02762010000300020, 2-s2.0-77953518821. 20512254

[50] Pérez-Losada M. , Cabezas P. , Castro-Nallar E. , and Crandall K. A. , Pathogen Typing in the Genomics Era: MLST and the Future of Molecular Epidemiology, Infection, Genetics and Evolution. (2013) 16, 38–53, 10.1016/j.meegid.2013.01.009, 2-s2.0-84875255868. 23357583

[51] Reiczigel J. , Marozzi M. , Fábián I. , and Rózsa L. , Biostatistics for Parasitologists – A Primer to Quantitative Parasitology, Trends in Parasitology. (2019) 35, no. 4, 277–281, 10.1016/j.pt.2019.01.003, 2-s2.0-85060760957. 30713051

[52] Felsenstein J. , Confidence Limits on Phylogenies: An Approach Using the Bootstrap, Evolution. (1985) 39, no. 4, 783–791, 10.1111/j.1558-5646.1985.tb00420.x. 28561359

[53] Nguyen L.-T. , Schmidt H. A. , von Haeseler A. , and Minh B. Q. , IQ-TREE: A Fast and Effective Stochastic Algorithm for Estimating Maximum-Likelihood Phylogenies, Molecular Biology and Evolution. (2015) 32, no. 1, 268–274, 10.1093/molbev/msu300, 2-s2.0-84922362345. 25371430 PMC4271533

[54] Felsenstein J. , Evolutionary Trees From DNA Sequences: A Maximum Likelihood Approach, Journal of Molecular Evolution. (1981) 17, no. 6, 368–376, 10.1007/BF01734359, 2-s2.0-0019797407. 7288891

[55] Rozas J. , Ferrer-Mata A. , and Sánchez-DelBarrio J. C. , et al.DnaSP 6: DNA Sequence Polymorphism Analysis of Large Data Sets, Molecular Biology and Evolution. (2017) 34, no. 12, 3299–3302, 10.1093/molbev/msx248, 2-s2.0-85041895435. 29029172

[56] Nei M. and Li W. H. , Mathematical Model for Studying Genetic Variation in Terms of Restriction Endonucleases, Proceedings of the National Academy of Sciences of the United States of America. (1979) 76, no. 10, 5269–5273, 10.1073/pnas.76.10.5269, 2-s2.0-0005814023. 291943 PMC413122

[57] Tajima F. , Evolutionary Relationship of DNA Sequences in Finite Populations, Genetics. (1983) 105, no. 2, 437–460, 10.1093/genetics/105.2.437. 6628982 PMC1202167

[58] Tajima F. , Statistical Method for Testing the Neutral Mutation Hypothesis by DNA Polymorphism, Genetics. (1989) 123, no. 3, 585–595, 10.1093/genetics/123.3.585. 2513255 PMC1203831

[59] Fu Y. X. and Li W. H. , Statistical Tests of Neutrality of Mutations, Genetics. (1993) 133, no. 3, 693–709, 10.1093/genetics/133.3.693. 8454210 PMC1205353

[60] Clement M. , Posada D. C. K. A. , and Crandall K. A. , TCS: A Computer Program to Estimate Gene Genealogies, Molecular Ecology. (2000) 9, no. 10, 1657–1659, 10.1046/j.1365-294x.2000.01020.x, 2-s2.0-0033771358. 11050560

[61] Leigh J. W. , Bryant D. , and Nakagawa S. , POPART: Full-Feature Software for Haplotype Network Construction, Methods in Ecology and Evolution. (2015) 6, no. 9, 1110–1116, 10.1111/2041-210X.12410, 2-s2.0-84941803316.

[62] Kong S. , Sánchez-Pacheco S. J. , and Murphy R. W. , On the use of Median-Joining Networks in Evolutionary Biology, Cladistics. (2016) 32, no. 6, 691–699, 10.1111/cla.12147, 2-s2.0-84951762898. 34753275

[63] Sánchez-Montes S. , Salceda-Sánchez B. , and Bermúdez S. E. , et al. *Rhipicephalus sanguineus* Complex in the Americas: Systematic, Genetic Diversity, and Geographic Insights, Pathogens. (2021) 10, no. 9, 10.3390/pathogens10091118. PMC8471700 34578151

[64] Ferreira A. S. , Ahmed A. , and Rocha T. , et al.Genetic Diversity of Pathogenic Leptospires From Wild, Domestic and Captive Host Species in Portugal, Transboundary and Emerging Diseases. (2020) 67, no. 2, 852–864, 10.1111/tbed.13409. 31677243

[65] Garcia-Lopez M. , Lorioux C. , and Soares A. , et al.Genetic Diversity of *Leptospira* Strains Circulating in Humans and Dogs in France in 2019–2021, Frontiers in Cellular and Infection Microbiology. (2023) 13, 10.3389/fcimb.2023.1236866, 1236866. 37662012 PMC10469827

[66] Garcia-Lopez M. , Lurier T. , and Bouilloud M. , et al.Prevalence, Genetic Diversity and Eco-Epidemiology of Pathogenic *Leptospira* Species in Small Mammal Communities in Urban Parks Lyon City, France, PLOS ONE. (2024) 19, no. 4, 10.1371/journal.pone.0300523, e0300523. 38598501 PMC11006123

[67] Dietrich M. , Gomard Y. , and Lagadec E. , et al.Biogeography of *Leptospira* in Wild Animal Communities Inhabiting the Insular Ecosystem of the Western Indian Ocean Islands and Neighboring Africa, Emerging Microbes & Infections. (2018) 7, no. 1, 1–12, 10.1038/s41426-018-0059-4, 2-s2.0-85044928436. 29615623 PMC5883017

[68] Pricemou P. S. , Soropogui B. , and Bérété F. , et al.Diversity of, *Leptospira*, Species and Their Rodent Reservoirs in the guinean Forest, Microorganisms. (2025) 13, no. 4, 10.3390/microorganisms13040833, 833. 40284669 PMC12029326

[69] Grillová L. , Robinson M. T. , and Chanthongthip A. , et al.Genetic Diversity of *Leptospira* Isolates in Lao PDR and Genome Analysis of an Outbreak Strain, PLOS Neglected Tropical Diseases. (2021) 15, no. 12, 10.1371/journal.pntd.0010076, e0010076. 34962921 PMC8746763

[70] Boonsilp S. , Thaipadungpanit J. , and Amornchai P. , et al.Peacock “A Single Multilocus Sequence Typing (MLST) Scheme for Seven Pathogenic *Leptospira* Species, PLoS Neglected Tropical Diseases. (2013) 7, no. 1, 10.1371/journal.pntd.0001954, 2-s2.0-84873493253, e1954. 23359622 PMC3554523

[71] Zarantonelli L. , Suanes A. , and Meny P. , et al.Isolation of Pathogenic *Leptospira* Strains From Naturally Infected Cattle in Uruguay Reveals High Serovar Diversity, and Uncovers a Relevant Risk for Human Leptospirosis, PLOS Neglected Tropical Diseases. (2018) 12, no. 9, 10.1371/journal.pntd.0006694, 2-s2.0-85054870940, e0006694. 30212451 PMC6136691

[72] Cerqueira G. M. , McBride A. J. , and Hartskeerl R. A. , et al.Bioinformatics Describes Novel Loci for High Resolution Discrimination of *Leptospira* Isolates, PLoS ONE. (2010) 5, no. 10, 10.1371/journal.pone.0015335, 2-s2.0-78149457700, e15335. 21124728 PMC2955542

[73] Hamond C. , Pestana C. P. , Medeiros M. A. , and Lilenbaum W. , Genotyping of *Leptospira* Directly in Urine Samples of Cattle Demonstrates a Diversity of Species and Strains in Brazil, Epidemiology and Infection. (2016) 144, no. 1, 72–75, 10.1017/S0950268815001363, 2-s2.0-84983119784. 26076668 PMC9507312

[74] Di Azevedo M. I. N. and Lilenbaum W. , An Overview on the Molecular Diagnosis of Animal Leptospirosis, Letters in Applied Microbiology. (2021) 72, no. 5, 496–508, 10.1111/lam.13442. 33332656

[75] Peláez Sanchez R. G. , Lopez J.Á. , Pereira M. M. , Arboleda M. , and Agudelo-Flórez P. , Genetic Diversity of *Leptospira* in Northwestern Colombia: First Report of *Leptospira santarosai* as a Recognised Leptospirosis Agent, Memórias do Instituto Oswaldo Cruz. (2016) 111, no. 12, 737–744, 10.1590/0074-02760160245, 2-s2.0-85005959238. 27982303 PMC5146737

[76] Jaeger L. H. , Pestana C. P. , Correia L. , Carvalho-Costa F. A. , Medeiros M. A. , and Lilenbaum W. , Novel MLST Sequence Types of Pathogenic *Leptospira* spp.: Opening the Black Box of Animal Leptospirosis in Brazil, Acta Tropica. (2019) 196, 135–141, 10.1016/j.actatropica.2019.05.025, 2-s2.0-85066049624. 31121146

[77] Loureiro A. P. , Jaeger L. H. , and Di Azevedo M. I. N. , et al.Molecular Epidemiology of *Leptospira noguchii* Reveals Important Insights Into a One Health Context, Transboundary and Emerging Diseases. (2019) 67, no. 1, 276–283, 10.1111/tbed.13349, 2-s2.0-85073975886. 31484225

[78] Haake D. A. and Zückert W. R. , The Leptospiral Outer Membrane. *Leptospira and Leptospirosis* , Current Topics in Microbiology and Immunology. (2015) 387, 187–221. 25388136 10.1007/978-3-662-45059-8_8 PMC4419373

[79] Santecchia I. , Ferrer M. F. , Vieira M. L. , Gómez R. M. , and Werts C. , Phagocyte Escape of *Leptospira*: the Role of TLRs and NLRs, Frontiers in Immunology. (2020) 11, 571816. 33123147 10.3389/fimmu.2020.571816 PMC7573490

[80] Hester S. E. , Park J. , Goodfield L. L. , Feaga H. A. , Preston A. , and Harvill E. T. , Horizontally Acquired Divergent O-Antigen Contributes to Escape From Cross-Immunity in the Classical Bordetellae, BMC Evolutionary Biology. (2013) 13, no. 209, 1–13, 10.1186/1471-2148-13-209, 2-s2.0-84884653417. 24067113 PMC3849452

[81] Pereira M. M. , Matsuo M. G. S. , and Bauab A. R. , et al.A Clonal Subpopulation of *Leptospira interrogans* Sensu Stricto is the Major Cause of Leptospirosis Outbreaks in Brazil, Journal of Clinical Microbiology. (2000) 38, no. 1, 450–452, 10.1128/JCM.38.1.450-452.2000. 10618140 PMC88748

[82] Stone N. E. , Hamond C. , and Clegg J. R. , et al.Host Population Dynamics Influence *Leptospira* spp. Transmission Patterns Among *Rattus norvegicus* in Boston, Massachusetts, US, PLOS Neglected Tropical Diseases. (2025) 19, no. 4, 10.1371/journal.pntd.0012966, e0012966. 40233129 PMC12047771

[83] Thaipadungpanit J. , Wuthiekanun V. , and Chierakul W. , et al.A Dominant Clone of *Leptospira interrogans* Associated With an Outbreak of Human Leptospirosis in Thailand, PLoS Neglected Tropical Diseases. (2007) 1, no. 1, 10.1371/journal.pntd.0000056, 2-s2.0-39449116981, e56. 17989782 PMC2041815

[84] Dutheil F. , Clinchamps M. , and Bouillon-Minois J.-B. , Bats, Pathogens, and Species Richness, Pathogens. (2021) 10, no. 2, 10.3390/pathogens10020098, 98. 33494226 PMC7909788

[85] Letko M. , Seifert S. N. , Olival K. J. , Plowright R. K. , and Munster V. J. , Bat-Borne Virus Diversity, Spillover and Emergence, Nature Reviews Microbiology. (2020) 18, no. 8, 461–471, 10.1038/s41579-020-0394-z. 32528128 PMC7289071

[86] Szentivanyi T. , McKee C. , Jones G. , and Foster J. T. , Trends in Bacterial Pathogens of Bats: Global Distribution and Knowledge Gaps, Transboundary and Emerging Diseases. (2023) 2023, no. 1, 1–17, 10.1155/2023/9285855, 9285855. PMC12017137 40303798

[87] Esteves S. B. , Gaeta N. C. , Batista J. M. N. , Dias R. A. , and Heinemann M. B. , *Leptospira* sp. Infection in Bats: A Systematic Review and Meta-Analysis, Transboundary and Emerging Diseases. (2022) 69, no. 5, e2456–e2473, 10.1111/tbed.14589. 35533065

[88] Suárez-Galaz A. R. , Panti-May J. A. , and Torres-Castro M. , Bats From Mexico and Bacteria Genera With Importance for Public or Animal Health, Tropical and Subtropical Agroecosystems. (2025) 28, no. 2.

[89] Silva-Ramos C. R. , Lemaitre G. P. , and Mejorano-Fonseca J. A. , et al.Molecular Evidence of *Leptospira* spp. Infection Among Household Dogs From 15 municipalities of the Department of Caldas, Colombia, Zoonoses and Public Health. (2025) 72, no. 2, 215–222, 10.1111/zph.13204. 39658809

[90] Verde R. S. , Di Azevedo M. I. N. , and Dias D. , et al.Bat-Associated Pathogenic *Leptospira* spp. From Forest Fragments in Southwestern Brazilian Amazonia, Transboundary and Emerging Diseases. (2024) 2024, no. 1, 10.1155/2024/6633866, 6633866. 40303138 PMC12017250

[91] Hamilton P. B. , Teixeira M. M. , and Stevens J. R. , The Evolution of *Trypanosoma Cruzi*: The ’Bat Seeding’ Hypothesis, Trends in Parasitology. (2012) 28, no. 4, 136–141, 10.1016/j.pt.2012.01.006, 2-s2.0-84859006932. 22365905

[92] McKee C. D. , Bai Y. , Webb C. T. , and Kosoy M. Y. , Bats are Key hosts in the Radiation of Mammal-Associated *Bartonella* Bacteria, Infection, Genetics and Evolution. (2021) 89, 10.1016/j.meegid.2021.104719, 104719. PMC10915969 33444855

[93] Martins G. , Loureiro A. P. , and Hamond C. , et al.First Isolation of *Leptospira noguchii* Serogroups Panama and Autumnalis From Cattle, Epidemiology and Infection. (2015) 143, no. 7, 1538–1541, 10.1017/S0950268814002416, 2-s2.0-84926296476. 25185756 PMC9507209

[94] Barragan V. , Chiriboga J. , and Miller E. , et al.High *Leptospira* Diversity in Animals and Humans Complicates the Search for Common Reservoirs of Human Disease in Rural Ecuador, PLOS Neglected Tropical Diseases. (2016) 10, no. 9, 10.1371/journal.pntd.0004990, 2-s2.0-84992092127, e0004990. 27622673 PMC5021363

[95] Aymée L. , Di Azevedo M. I. N. , and de Melo J. D. S. L. , et al. *Leptospira noguchii* Associated to Reproductive Disease in Ruminants, Transboundary and Emerging Diseases. (2022) 69, no. 5, 3103–3108, 10.1111/tbed.14377. 34741442

[96] Kremer F. S. , Eslabão M. R. , and Provisor M. , et al.Draft Genome Sequences of *Leptospira santarosai* Strains U160, U164, and U233, Isolated From Asymptomatic Cattle, Genome Announcements. (2015) 3, no. 4, 10–1128, 10.1128/genomeA.00910-15, 2-s2.0-85008452744. PMC4536688 26272577

[97] Aymée L. , Di Azevedo M. I. N. , Borges A. L. D. S. B. , Carvalho-Costa F. A. , and Lilenbaum W. , *Leptospira* spp. Strains Associated With Bovine Genital Leptospirosis (BGL), Microbial Pathogenesis. (2022) 173, 10.1016/j.micpath.2022.105841, 105841. 36309182

[98] Di Azevedo M. I. N. , Soares A. C. D. R. , Ezepha C. , Carvalho-Costa F. A. , Vieira A. S. , and Lilenbaum W. , Genetic Characterization and Zoonotic Potential of, *Leptospira interrogans*, Identified in Small Non-Flying Mammals From Southeastern Atlantic Forest, Brazil, Tropical Medicine and Infectious Disease. (2025) 10, no. 3, 10.3390/tropicalmed10030062, 62. 40137816 PMC11945321

[99] Ramos-Vázquez J. R. , Sánchez-Montes S. , and Esparza-González S. C. , et al.Isolation and Molecular Identification of *Leptospira santarosai* and *Leptospira interrogans* in Equines From Eastern Mexico, Acta Tropica. (2024) 256, 10.1016/j.actatropica.2024.107242, 107242. 38782111

[100] Aymée L. , Gregg W. R. R. , and Loureiro A. P. , et al.Bovine Genital Leptospirosis and Reproductive Disorders of Live Subfertile Cows Under Field Conditions, Veterinary Microbiology. (2021) 261, 10.1016/j.vetmic.2021.109213, 109213. 34481272

[101] Putz E. J. and Nally J. E. , Investigating the Immunological and Biological Equilibrium of Reservoir Hosts and Pathogenic *Leptospira*: Balancing the Solution to an Acute Problem?, Frontiers in Microbiology. (2020) 11, 2005. 32922382 10.3389/fmicb.2020.02005 PMC7456838

[102] Jorge S. , Hartleben C. P. , and Seixas F. K. , et al. *Leptospira borgpetersenii* From Free-Living White-Eared Opossum (*Didelphis albiventris*): First Isolation in Brazil, Acta Tropica. (2012) 124, no. 2, 147–151, 10.1016/j.actatropica.2012.07.009, 2-s2.0-84865725507. 22897870

[103] Horta M. C. , Ragozo A. M. A. , and Casagrande R. A. , et al.Occurrence of Anti-Toxoplasma Gondii, Neospora Caninum and *Leptospira* spp. Antibodies in Opossums (Didelphis spp.) in São Paulo State, Brazil, Brazilian Journal of Veterinary Research and Animal Science. (2016) 53, no. 3, 1–9, 10.11606/issn.1678-4456.bjvras.2016.110381, 2-s2.0-85032449831.

[104] Fornazari F. , Langoni H. , Marson P. M. , Nóbrega D. B. , and Teixeira C. R. , *Leptospira* Reservoirs Among Wildlife in Brazil: Beyond Rodents, Acta Tropica. (2018) 178, 205–212, 10.1016/j.actatropica.2017.11.019, 2-s2.0-85036592809. 29197499

[105] Suárez-Galaz A. , Reyes-Novelo E. , and Cruz-Romero A. , et al.The Relationship Between the Spatial Occurrence of *Leptospira* Exposed Animals and the Characteristics of the Peridomiciles They Inhabit in a Locality of Southeastern Mexico, Pathogens. (2024) 13, no. 12, 1037. 39770297 10.3390/pathogens13121037 PMC11728841

[106] Boey K. , Shiokawa K. , Rajeev S. , and Day N. P. , *Leptospira* Infection in Rats: A Literature Review of Global Prevalence and Distribution, PLOS Neglected Tropical Diseases. (2019) 13, no. 8, 10.1371/journal.pntd.0007499, 2-s2.0-85071282321, e0007499. 31398190 PMC6688788

[107] Colombo V. C. , Gamietea I. , Loffler S. G. , Brihuega B. F. , and Beldomenico P. M. , New Host Species for *Leptospira borgpetersenii* and *Leptospira interrogans* Serovar Copenhageni, Veterinary Microbiology. (2018) 215, 90–92, 10.1016/j.vetmic.2018.01.007, 2-s2.0-85041483867. 29426412

[108] Jaeger L. H. , Pestana C. P. , Carvalho-Costa F. A. , Medeiros M. A. , and Lilenbaum W. , Characterization of the Clonal Subpopulation Fiocruz L1-130 of *Leptospira interrogans* in Rats and Dogs From Brazil, Journal of Medical Microbiology. (2018) 67, no. 9, 1361–1367, 10.1099/jmm.0.000806, 2-s2.0-85053000082. 30059000

[109] Lei B. R. , Olival K. J. , and Small P. L. C. , Contrasting Patterns in Mammal–Bacteria Coevolution: *Bartonella* and *Leptospira* in Bats and Rodents, PLOS Neglected Tropical Diseases. (2014) 8, no. 3, 10.1371/journal.pntd.0002738, 2-s2.0-84897416937, e2738. 24651646 PMC3961187

[110] Davignon G. , Cagliero J. , and Guentas L. , et al.Leptospirosis: Toward a Better Understanding of the Environmental Lifestyle of *Leptospira* , Frontiers in Water. (2023) 5, 10.3389/frwa.2023.1195094, 1195094.

[111] Di Azevedo M. I. N. , Aymée L. , Borges A. L. D. S. B. , and Lilenbaum W. , Molecular Epidemiology of Pathogenic *Leptospira* spp. Infecting Dogs in Latin America, Animals. (2023) 13, no. 15, 10.3390/ani13152422, 2422. 37570231 PMC10417440

[112] Guedes I. B. , Oliveira de Souza G. , and Fernandes de Paula Castro J. , et al.Identification of Pathogenic *Leptospira* Species in the Urogenital Tract of Water Buffaloes (*Bubalus bubalis*) From the Amazon River Delta Region, Brazil, Frontiers in Veterinary Science. (2020) 7, 10.3389/fvets.2020.00269, 269. 32478114 PMC7241293

[113] Di Azevedo M. I. N. , Kremer F. , and Ezepha C. , et al.Comparative Genomics of *Leptospira santarosai* Reveals Genomic Adaptations in Bovine Genital Strains, Frontiers in Microbiology. (2025) 15, 10.3389/fmicb.2024.1517151, 1517151. 39839101 PMC11747425

[114] Vieira A. S. , Pinto P. S. , and Lilenbaum W. , A Systematic Review of Leptospirosis on Wild Animals in Latin America, Tropical Animal Health and Production. (2018) 50, no. 2, 229–238, 10.1007/s11250-017-1429-y, 2-s2.0-85030153775. 28967042

[115] Bernheim A. , Cury J. , and Poirier E. Z. , The Immune Modules Conserved Across the Tree of Life: Towards a Definition of Ancestral Immunity, PLOS Biology. (2024) 22, no. 7, 10.1371/journal.pbio.3002717, e3002717. 39008452 PMC11249213

[116] Sheppard S. K. , Guttman D. S. , and Fitzgerald J. R. , Population Genomics of Bacterial Host Adaptation, Nature Reviews Genetics. (2018) 19, no. 9, 549–565, 10.1038/s41576-018-0032-z, 2-s2.0-85049552294. 29973680

[117] Barber M. F. and Fitzgerald J. R. , Mechanisms of Host Adaptation by Bacterial Pathogens, FEMS Microbiology Reviews. (2024) 48, no. 4, fuae019 10.1093/femsre/fuae019. PMC11308195 39003250

[118] Viana Martín D. , Selva L. , Penadés M. , and Corpa J. M. , Screening of Virulence Genes in *Staphylococcus aureus* Isolates From Rabbits, World Rabbit Science. (2015) 23, no. 3, 185–195, 10.4995/wrs.2015.3961, 2-s2.0-84944387846.

[119] Afreen N. , Naqvi I. H. , Broor S. , Ahmed A. , and Parveen S. , Phylogenetic and Molecular Clock Analysis of Dengue Serotype 1 and 3 From New Delhi, India, PLOS ONE. (2015) 10, no. 11, 10.1371/journal.pone.0141628, 2-s2.0-84951185311, e0141628. 26536458 PMC4633233

[120] Arellano-Llamas R. , Alfaro-Ruiz L. , and Arriaga Canon C. , et al.Molecular Features of Influenza A (H1N1) pdm09 Prevalent in Mexico During Winter Seasons 2012–2014, PLOS ONE. (2017) 12, no. 7, 10.1371/journal.pone.0180419, 2-s2.0-85022340584, e0180419. 28692701 PMC5503254

[121] Bhattacharjee U. , Chakrabarti A. K. , Kanungo S. , and Dutta S. , Evolutionary Dynamics of Influenza A/H1N1 Virus Circulating in India From 2011 to 2021, Infection, Genetics and Evolution. (2023) 110, 10.1016/j.meegid.2023.105424, 105424. 36913995

[122] Eldholm V. , Monteserin J. , and Rieux A. , et al.Four Decades of Transmission of a Multidrug-Resistant *Mycobacterium tuberculosis* Outbreak Strain, Nature Communications. (2015) 6, no. 1, 10.1038/ncomms8119, 2-s2.0-84929192008, 7119. PMC4432642 25960343

[123] Folkvardsen D. B. , Norman A. , Andersen Å.B. , Rasmussen E. M. , Jelsbak L. , and Lillebaek T. , Genomic Epidemiology of a Major, *Mycobacterium tuberculosis*, Outbreak: Retrospective Cohort Study in a Low-Incidence Setting Using Sparse Time-Series Sampling, The Journal of Infectious Diseases. (2017) 216, no. 3, 366–374, 10.1093/infdis/jix298, 2-s2.0-85029550829. 28666374

[124] Kühnert D. , Coscolla M. , and Brites D. , et al.Tuberculosis Outbreak Investigation Using Phylodynamic Analysis, Epidemics. (2018) 25, 47–53, 10.1016/j.epidem.2018.05.004, 2-s2.0-85048591034. 29880306 PMC6227250

[125] Agampodi S. B. , Moreno A. C. , Vinetz J. M. , and Matthias M. A. , Utility and Limitations of Direct Multi-Locus Sequence Typing on qPCR-Positive Blood to Determine Infecting *Leptospira* Strain, The American Society of Tropical Medicine and Higiene. (2013) 88, no. 1, 184–185, 10.4269/ajtmh.2012.12-0526, 2-s2.0-84872318834. PMC3541733 23208890

[126] Varni V. , Ruybal P. , and Lauthier J. J. , et al.Reassessment of MLST Schemes for *Leptospira* spp. Typing Worldwide, Infection, Genetics and Evolution. (2014) 22, 216–222, 10.1016/j.meegid.2013.08.002, 2-s2.0-84896721654. 23932960

[127] Weiss S. , Menezes A. , and Woods K. , et al.An Extended Multilocus Sequence Typing (MLST) Scheme for Rapid Direct Typing of *Leptospira* From Clinical Samples, PLOS Neglected Tropical Diseases. (2016) 10, no. 9, 10.1371/journal.pntd.0004996, 2-s2.0-84992089297, e0004996. 27654037 PMC5031427

[128] Chiani Y. , Jacob P. , and Varni V. , et al.Isolation and Clinical Sample Typing of Human Leptospirosis Cases in Argentina, Infection, Genetics and Evolution. (2016) 37, 245–251. 10.1016/j.meegid.2015.11.033 26658064

[129] Ahmed A. , Thaipadungpanit J. , and Boonsilp S. , et al.Peacock “Comparison of Two Multilocus Sequence Based Genotyping Schemes for *Leptospira* Species, PLoS Neglected Tropical Diseases. (2011) 5, no. 11, 10.1371/journal.pntd.0001374, 2-s2.0-82555203056, e1374. 22087342 PMC3210738

[130] Barragan V. , Olivas S. , Keim P. , and Pearson T. , Critical Knowledge Gaps in Our Understanding of Environmental Cycling and Transmission of *Leptospira* spp., Applied and Environmental Microbiology. (2017) 83, no. 19, e01190–e01117, 10.1128/AEM.01190-17, 2-s2.0-85029551552. 28754706 PMC5601346

[131] Mwachui M. A. , Crump L. , Hartskeerl R. , Zinsstag J. , Hattendorf J. , and Small P. L. C. , Environmental and Behavioural Determinants of Leptospirosis Transmission: A Systematic Review, PLOS Neglected Tropical Diseases. (2015) 9, no. 9, 10.1371/journal.pntd.0003843, 2-s2.0-84943196151, e0003843. 26379035 PMC4574979

[132] Bradley E. A. and Lockaby G. , Leptospirosis and the Environment: A Review and Future Directions, Pathogens. (2023) 12, no. 9, 1167. 37764975 10.3390/pathogens12091167 PMC10538202

[133] Grassly N. C. , Shaw A. G. , and Owusu M. , Global Wastewater Surveillance for Pathogens With Pandemic Potential: Opportunities and Challenges, The Lancet Microbe. (2025) 6, no. 1, 10.1016/j.lanmic.2024.07.002, 100939. 39222653

[134] Byrnes E. J. , Li W. , and Lewit Y. , et al.Emergence and Pathogenicity of Highly Virulent *Cryptococcus gattii* Genotypes in the Northwest United States, PLoS Pathogens. (2010) 6, no. 4, 10.1371/journal.ppat.1000850, e1000850. 20421942 PMC2858702

[135] Vanderkooi O. G. , Church D. L. , MacDonald J. , Zucol F. , Kellner J. D. , and Borrow R. , Community-Based Outbreaks Invulnerable Populations of Invasive Infections Caused by *Streptococcus pneumoniae* Serotypes 5 and 8 in Calgary, Canada, PLoS ONE. (2011) 6, no. 12, 10.1371/journal.pone.0028547, 2-s2.0-84455161879, e28547. 22216100 PMC3246448

[136] Matsunari O. , Shiota S. , and Suzuki R. , et al.Association Between *Helicobacter pylori* Virulence Factors and Gastroduodenal Diseases in Okinawa, Japan, Journal of Clinical Microbiology. (2012) 50, no. 3, 876–883, 10.1128/JCM.05562-11, 2-s2.0-84857424756. 22189111 PMC3295155

[137] Diard M. and Hardt W.-D. , Evolution of Bacterial Virulence, FEMS Microbiology Reviews. (2017) 41, no. 5, 679–697, 10.1093/femsre/fux023, 2-s2.0-85031897662. 28531298

[138] Urwin R. , Russell J. E. , Thompson E. A. , Holmes E. C. , Feavers I. M. , and Maiden M. C. J. , Distribution of Surface Protein Variants Among Hyperinvasive Meningococci: Implications for Vaccine Design, Infection and Immunity. (2004) 72, no. 10, 5955–5962, 10.1128/IAI.72.10.5955-5962.2004, 2-s2.0-4644237146. 15385499 PMC517544

[139] Bambini S. , Muzzi A. , Olcen P. , Rappuoli R. , Pizza M. , and Comanducci M. , Distribution and Genetic Variability of Three Vaccine Components in a Panel of Strains Representative of the Diversity of Serogroup B Meningococcus, Vaccine. (2009) 27, no. 21, 2794–2803, 10.1016/j.vaccine.2009.02.098, 2-s2.0-64449085566. 19428890

[140] Racloz V. N. and Luiz S. J. D. , The Elusive Meningococcal Meningitis Serogroup: A Systematic Review of Serogroup B Epidemiology, BMC Infectious Diseases. (2010) 10, no. 1, 10.1186/1471-2334-10-175, 2-s2.0-77953594002, 175. 20565757 PMC2894839

[141] Hanage W. P. , Bishop C. J. , and Lee G. M. , et al.Clonal Replacement Among 19A *Streptococcus pneumoniae* in Massachusetts, Prior to 13 valent Conjugate Vaccination, Vaccine. (2011) 29, no. 48, 8877–8881, 10.1016/j.vaccine.2011.09.075, 2-s2.0-82455167938. 21964059 PMC3221484

[142] Pichon B. , Bennett H. V. , Efstratiou A. , Slack M. P. E. , and George R. C. , Genetic Characteristics of Pneumococcal Disease in Elderly Patients Before Introducing the Pneumococcal Conjugate Vaccine, Epidemiology and Infection. (2009) 137, no. 7, 1049–1056, 10.1017/S0950268808001787, 2-s2.0-67650492421. 19161642

[143] Adetifa I. M. , Antonio M. , and Okoromah C. A. , et al.Pre-Vaccination Nasopharyngeal Pneumococcal Carriage in a Nigerian Population: Epidemiology and Population Biology, PLoS ONE. (2012) 7, no. 1, 10.1371/journal.pone.0030548, 2-s2.0-84856158775, e30548. 22291984 PMC3265474

[144] Sheppard S. K. , Colles F. , and Richardson J. , et al.Host Association of *Campylobacter* Genotypes Transcends Geographic Variation, Applied and Environmental Microbiology. (2010) 76, no. 15, 5269–5277, 10.1128/AEM.00124-10, 2-s2.0-77955568382. 20525862 PMC2916502

[145] Hotchkiss E. J. , Hodgson J. C. , Lainson F. A. , and Zadoks R. N. , Multilocus Sequence Typing of a Global Collection of *Pasteurella multocida* Isolates From Cattle and Other Host Species Demonstrates Niche Association, BMC Microbiology. (2011) 11, no. 1, 10.1186/1471-2180-11-115, 2-s2.0-80052546059, 115. 21612618 PMC3120644

